# A composite score using quantitative magnetic resonance cholangiopancreatography predicts clinical outcomes in primary sclerosing cholangitis

**DOI:** 10.1016/j.jhepr.2023.100834

**Published:** 2023-06-29

**Authors:** Raj Vuppalanchi, Vijay Are, Alison Telford, Liam Young, Sofia Mouchti, Carlos Ferreira, Carla Kettler, Mark Gromski, Fatih Akisik, Naga Chalasani

**Affiliations:** 1 Division of Gastroenterology and Hepatology, Indiana University School of Medicine, Indianapolis, IN, USA; 2 Perspectum Ltd., Oxford, UK; 3 Department of Radiology, Indiana University School of Medicine, Indianapolis, IN, USA

**Keywords:** MRCP, Non-invasive, Quantitative MRI, PSC, Prognostic biomarkers

## Abstract

**Background & Aims:**

Magnetic resonance cholangiopancreatography (MRCP) for evaluation of biliary disease currently relies on subjective assessment with limited prognostic value because of the lack of quantitative metrics. Artificial intelligence-enabled quantitative MRCP (MRCP+) is a novel technique that segments biliary anatomy and provides quantitative biliary tree metrics. This study investigated the utility of MRCP+ as a prognostic tool for the prediction of clinical outcomes in primary sclerosing cholangitis (PSC).

**Methods:**

MRCP images of patients with PSC were post-processed using MRCP+ software. The duration between the MRCP and clinical event (liver transplantation or death) was calculated. Survival analysis and stepwise Cox regression were performed to investigate the optimal combination of MRCP+ metrics for the prediction of clinical outcomes. The resulting risk score was validated in a separate validation cohort and compared with an existing prognostic score (Mayo risk score).

**Results:**

In this retrospective study, 102 patients were included in a training cohort and a separate 50 patients formed a validation cohort. Between the two cohorts, 34 patients developed clinical outcomes over a median duration of 3 years (23 liver transplantations and 11 deaths). The proportion of bile ducts with diameter 3–5 mm, total bilirubin, and aspartate aminotransferase were independently associated with transplant-free survival. Combined as a risk score, the overall discriminative performance of the MRCP+ risk score (M+BA) was excellent; area under the receiver operator curve 0.86 (95% CI: 0.77, 0.95) at predicting clinical outcomes in the validation cohort with a hazard ratio 5.8 (95% CI: 1.5, 22.1). This was superior to the Mayo risk score.

**Conclusions:**

A composite score combining MRCP+ with total bilirubin and aspartate aminotransferase (M+BA) identified PSC patients at high risk of liver transplantation or death. Prospective studies are warranted to evaluate the clinical utility of this novel prognostic tool.

**Impact and Implications:**

Primary sclerosis cholangitis (PSC) is a disease of the biliary tree where inflammation and fibrosis cause areas of narrowing (strictures) and expansion (dilatations) within the biliary ducts leading to liver failure and/or cancer (cholangiocarcinoma). In this study, we demonstrate that quantitative assessment of the biliary tree can better identify patients with PSC who are at high risk of either death or liver transplantation than a current blood-based risk score (Mayo risk score).

## Highlights

• Many people with PSC will require a liver transplant to extend their life but current stratification methods struggle to identify all patients at high-risk of progression to this stage, especially at the early stages of disease.
• A novel post-processing technique (MRCP+) enables quantification of magnetic resonance cholangiopancreatography (MRCP) data, providing quantitative assessments of biliary anatomy and abnormality for enhanced monitoring of biliary diseases such as PSC.
• A risk score comprised of the proportion of the bile ducts with diameter 3–5 mm, total bilirubin and aspartate aminotransferase (AST) was highly predictive of clinical outcomes in a validation cohort (hazard ratio = 5.8), outperforming the Mayo risk score.

## Graphical abstract

**Figure:**
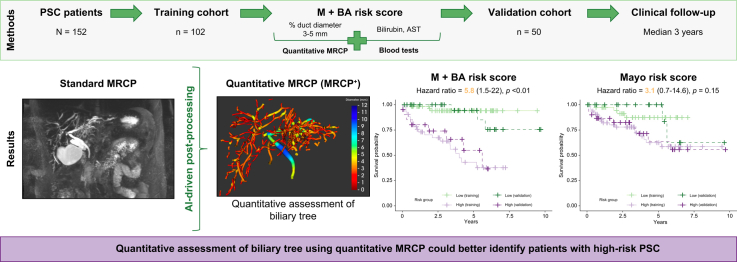

## Introduction

Primary sclerosing cholangitis (PSC) is a chronic and progressive cholestatic liver disease characterised by multi-focal stricturing throughout the biliary tree.[1], [2], [3], [4] Currently, liver transplantation is the only lifesaving intervention. Although there are no effective pharmacotherapies approved, accurately characterising the disease course is of critical importance for developing new, effective, and affordable treatments. Identifying those at highest risk of poor outcomes is an integral part of the clinical assessment to stratify those at the most need of intervention with endoscopic retrograde cholangiopancreatography (ERCP) and, or referral to liver transplantation in a timely manner.^3^^,^^4^ Opportunities for intervention are made more challenging by the rareness of symptoms in early-stage PSC alongside a lack of effective biomarkers.[3], [4], [5] Although several risk scores and biomarkers have been developed to estimate rates of disease progression,[6], [7], [8], [9], [10], [11], [12] their performance remains limited^13^ and relies heavily upon biochemical markers which may fluctuate or lag behind parenchymal and ductal changes.^14^

**Fig. 1:**
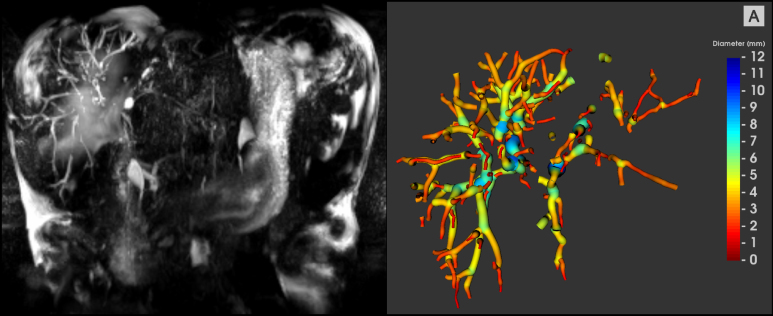
Example MRCP maximum intensity projection and MRCP+ model from a patient with large duct PSC. In the MRCP+ model, shown on the right, the diameter of each duct is shown by the colourmap and hence regions of dilatation can be clearly seen (blue). Quantitative analyses show a total biliary tree volume of 29.2 ml, 133 modelled ducts, 16 potential strictures, 50 potential dilations, 2538.7 mm of total length of ducts with a 438.7 mm of total length of strictures and dilatations. MRCP, magnetic resonance cholangiopancreatography.

Current clinical guidelines for the diagnosis and management of PSC are based on the cholangiographic or histological features of sclerosing cholangitis in the absence of identifiable causes of secondary sclerosing cholangitis.^3^^,^^4^ Although ERCP is the traditional gold standard, there is an associated risk of pancreatitis or cholangitis.^3^^,^^4^ Given that around 60% of patients with large duct PSC show evidence of radiological progression over a 4-year follow up,^15^ non-invasive imaging-based investigations such as magnetic resonance cholangiopancreatography (MRCP) are now recommended in patients who have new or changing symptoms, or evolving abnormalities in laboratory investigations.^3^^,^^4^^,^^16^ Despite the widespread use of MRCP, there remain important limitations, including poor bile duct depiction as well as subjective assessments and unstandardised acquisition protocols. Recently, artificial intelligence-enabled (AI-enabled) software has been developed that enhances conventional MRCP to produce quantitative MRCP (MRCP+™, Perspectum Ltd, Oxford, UK). The software produces a 3D model of the biliary tree which aids visualisation, but more importantly, provides novel quantitative measures for the direct assessment of ductal anatomy.^17^ Because of the heterogeneity of bile duct alterations observed in PSC, the adoption of quantitative evaluation of MRCP images has the potential to improve diagnostic performance, reduce clinician burden and sensitively monitor ductal change over time.^18^ MRCP+ calculates quantitative 3D biliary system models from historical MRCP images, enabling the measurement of bile duct widths and automatic detection of regions of variation of duct widths. It also allows for regional volumetric analysis of the biliary tree, pancreatic duct, and gallbladder (Fig. 1).

In recent studies, multiple MRCP+ parameters were closely associated with biochemical scoring systems and magnetic resonance elastography (MRE) and demonstrated the potential to act as a risk stratification tool in PSC.^17^^,^^19^ Until recently, it was unknown how to combine multiple predictive metrics with other measures to provide a clear probability of an outcome. This approach of combining a panel of relevant markers, which individually capture aspects of disease may prove useful in providing a more granular assessment. However, as highlighted by Moons *et al.*^20^ deriving a prognostic model is challenging, requiring multiple studies to first develop the new score and assess its promise using internal validation, then validate the new score in external cohorts, before finally assessing the impact of the prognostic model on clinical practice.[20], [21], [22] Two recent studies have now taken the first steps of developing a prognostic model. Cristoferi *et al.*^23^ and Cazzagon *et al.*^24^ independently developed prognostic models that contained quantitative MRCP metrics. In their study of 87 patients with PSC Cristoferi *et al.*^23^ found that combining the count of number of strictures with a measurement of spleen size enabled the discrimination of survival. Meanwhile, Cazzagon *et al.*^24^ found in their study of 77 patients with PSC that median duct diameter was significantly associated with patient outcomes.

Building on these encouraging studies, this new work aimed to both develop and validate a prognostic model, using MRCP+, to predict clinical outcomes, including death and liver transplant, in patients with PSC. We then aimed to compare this new model to the revised Mayo risk score.^12^ In doing so, we evaluate the promise of a risk classifier based on a data-driven combination of MRCP+ and blood tests to predict death or liver transplant among patients with PSC.

## Patients and methods

### Design and study participants

This retrospective study consisted of data initially collected after informed consent as part of prospective databases of PSC patients undergoing ERCP and those attending outpatient hepatology clinics, along with retrospective liver transplant registry data obtained through Organ Transplant Tracking Record Systems at Indiana University Academic Health Center, Indianapolis, IN, USA. Study participants included in the current analysis were those who underwent MRCP and standard biochemical tests required to calculate the revised Mayo risk score.^12^ The baseline visits of individuals in this study occurred between June 2009 and August 2019. All participants were followed up for liver transplant and death. This study was approved by the Institutional Review Board at Indiana University School of Medicine. For the current study, patients who had either incomplete MRCP+ metrics or blood biochemistry were excluded. Death date was confirmed through electronic health records or the national death index.^25^

### Imaging acquisition

Scans were performed on 1.5 Tesla (T) and 3T clinical magnetic resonance imaging (MRI) scanners available as part of participants' routine clinical evaluation. Heavily T2-weighted MRCP images were acquired using 3D multi-shot fast/turbo spin echo acquisitions with long echo train lengths and short echo spacing to generate 3D volumetric images. Parameters used include an echo time (TE) range from 347 ms to 866 ms and 40–160 contiguous slices with 1–1.2 mm slice thickness. The pixel resolution of the acquired data varied from 0.4 mm × 0.4 mm to 1.2 mm × 1.2 mm. 3D MRCP images were acquired in a coronal oblique orientation to cover the largest extent of the biliary tree and pancreatic duct. Respiratory gating with navigator tracking was used for data acquisition. The expiration phase of the breathing cycle was defined as the period for image acquisition, so the repetition time (TR) varied with the breathing rate. Fat suppression techniques were used to suppress signals from fat, and parallel imaging techniques were used to reduce scanning time.

### Imaging processing

Archived MRCP images were assigned a unique study identifier after removing protected health information and then uploaded. These files were then processed with MRCP+ software, which processes 3D MRCP acquisitions using AI-driven pathfinding algorithms and tubular enhancement techniques to derive a quantitative parametric model of the biliary tree and pancreatic ducts.^26^ Ninety-two quantitative MRCP metrics that characterise biliary tree structure and include measures of volume and duct length, as well as numbers of strictures and dilatations, were calculated from the MRCP+ models. Detailed information regarding MRCP+ is provided within the Supplementary material. The resulting models were assessed for quality by a trained analyst. An example of MRCP+ is shown in Fig. 1.

### Statistical analysis

Analyses were performed using R software version 4.0.2 (R Foundation for Statistical Computing, Vienna, Austria). Descriptive statistics are presented for all participants, median with IQR used to describe continuous variables, and frequency and percentage for categorical variables. The distribution of the continuous variables between the presence and absence of clinical outcome groups was compared with the Wilcoxon Sum of Ranks; the Chi-square test was used to compare the distribution of the presence of a categorical variable.

The prognostic model was constructed and validated using a split-sample approach. Briefly, the cohort was randomly split into a training and validation cohort with a ratio of 3:1. Next, the quantitative MRCP metrics were filtered to ensure only metrics with previously reported good or excellent reproducibility (intraclass correlation [ICC] >0.6) were considered for inclusion in the prognostic model.^27^ Biochemical metrics, serum aspartate aminotransferase (AST), alanine aminotransferase (ALT), albumin, platelet count, total bilirubin, alkaline phosphatase (ALP), were log-transformed as previously described by de Vries *et al.*^28^

A stepwise variable selection approach was performed on the training dataset with the reduced set of quantitative MRCP and biochemical metrics input to identify the optimal combination of metrics for the prognostic model.^29^ A strict variance inflation factor of 2.5 was chosen to reduce the negative impact of multicollinearity on the model.^30^ Discrimination of the model in both the training and validation data sets was assessed through Harwell’s C-statistic.

Using the derived model, an M+BA risk score was calculated for each patient. Overall diagnostic accuracy of the M+BA risk score was estimated by the area under the receiver operator curve (AUROC), and an optimal cut-off to categorise individuals as high risk and low risk was derived using Youden’s index from the training cohort. In line with previous studies, a single threshold of zero was applied to the revised Mayo risk score to categorise individuals as high-risk and low-risk.^12^^,^^31^

The categorisations defined by the M+BA risk score and the revised Mayo risk score were compared by considering the hazard ratios for those classified as high risk by both risk scores, respectively. Kaplan–Meier curves were plotted to present the cumulative probability of transplant-free survival since baseline in both the training and validation cohorts. Akaike’s Information Criteria (AIC) was used to assess which risk score provided a better fit for explaining transplant-free survival. The prognostic accuracy of each for predicting death and/or liver transplant in patients with PSC in the short term (≤3 years) and long term (>3 years) was also assessed in the validation cohort.

To investigate whether an increased sample size would reveal any additional signal, we conducted a secondary, exploratory analysis in which both training and validation cohorts were pooled, and the analysis (excluding validation steps) were repeated. This secondary analysis is detailed within the Supplementary material.

## Results

**Fig. 2:**
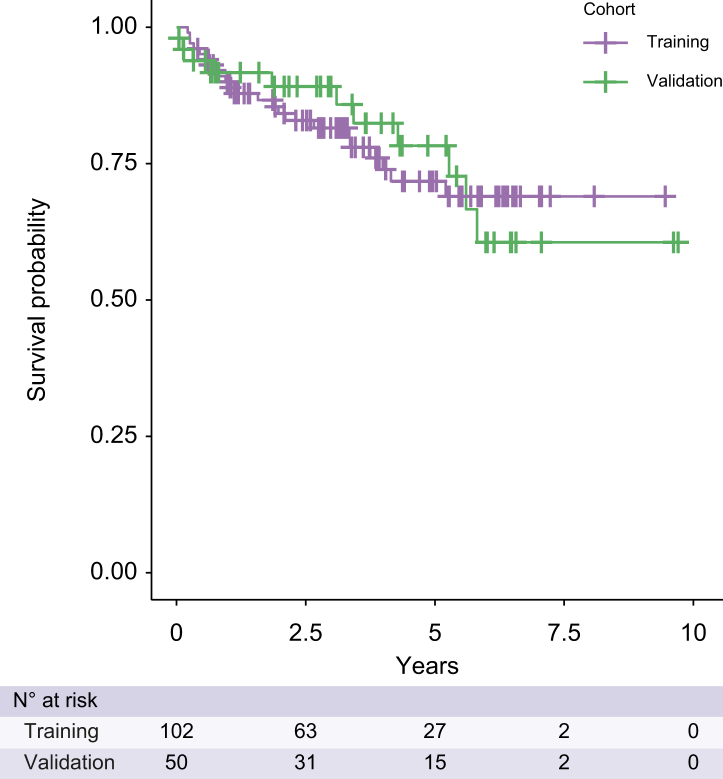
Kaplan–Meier survival curves for training and validation cohorts.

Records of 173 patients with PSC with multiple clinic visits were available for review. After applying the exclusion criteria, 152 participants with baseline data were included in the analysis, with 102 patients included in the training cohort (63% male; median BMI of 27 kg/m^2^; 84% Caucasian) and 50 forming a validation dataset (76% male; median BMI of 26 kg/m^2^; 82% Caucasian). No significant differences in demographics were seen between patients in the test and validation cohorts (Table 1). In the training and validation cohorts, 14 and 4 patients had a liver transplant and 9 and 7 patients died respectively with median transplant-free survival of 3 years for both cohorts (Fig. 2). The percentage of ducts with a diameter between 3 and 5 mm, the Mayo risk score, average albumin, bilirubin, AST, ALP, international normalised ratio, and Fibrosis-4 (FIB4), were significantly different between the group of participants who experienced the clinical event and those who did not (Table S1) but were comparable between the training and validation cohorts.

**Table 1:** Patient characteristics.

	All	Training cohort	Validation cohort	*p* value∗
N (%)	152 (100)	102 (100.0)	50 (100.0)	
Age (years); median (IQR)	48 (32–63)	48 (33–61)	51 (30–66)	>0.9
Sex (males); n (%)	102 (67)	64 (63)	38 (76)	0.5
BMI (kg/m^2^); median (IQR)	26.6 (23.2–30.0)	27.1 (23.8–31.0)	25.6 (22.3–28.4)	0.4
Ethnicity; n (%)				>0.9
Caucasian	122 (84)	85 (84)	37 (82)	
African-American	21 (14)	14 (14)	7 (16)	
Other	3 (2.1)	2 (2)	1 (2.2)	
Alcohol abuse; n (%)	16 (11)	13 (13)	3 (6.2)	0.7
Cholecystectomy at baseline; n (%)	13 (8.6)	9 (8.8)	4 (8.0)	>0.9
Type of IBD; n (%)				>0.9
Ulcerative colitis	85 (76)	62 (77)	23 (74)	
Crohn’s disease	26 (23)	18 (22)	8 (26)	
Indeterminate colitis	1 (0.9)	1 (1.2)	0 (0.0)	
UDCA class; n (%)				>0.9
Non-user	63 (43)	40 (41)	23 (46)	
Regular dose UDCA	76 (51)	52 (53)	24 (48)	
High dose UDCA	9 (6.1)	6 (6.1)	3 (6.0)	

Data are presented as mean (SD) or median (IQR) for continuous variables, and as numbers and percentages (%) for categorical data.

BMI, body mass index; IBD, inflammatory bowel disease; IQR, interquartile range; UDCA, ursodeoxycholic acid.

∗ Bonferroni corrected *p* value.

### Prediction of outcomes

**Fig. 3:**
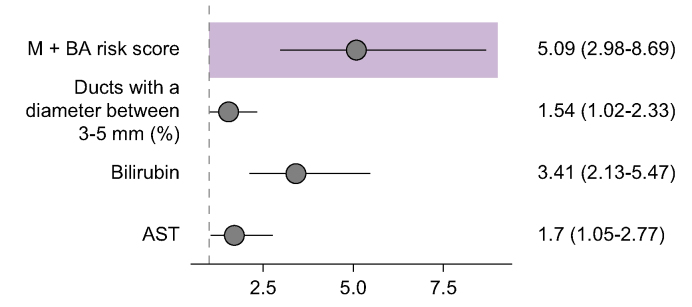
Forest plot. Shaded box: hazard ratio and corresponding 95% CI of the composite M+BA risk score. Below shaded box: hazard ratios and corresponding 95% CI of the quantitative MRCP and blood metrics chosen by the Stepwise Cox regression model. All metrics were standardised as z-scores. AST, aspartate aminotransferase; MRCP, magnetic resonance cholangiopancreatography.

The stepwise regression method for Cox’s Proportional Hazard model returned the risk score of quantitative MRCP and blood metrics (M+BA risk score). The three M+BA model metrics (Fig. 3) were: proportion of bile ducts with diameter 3–5 mm (%), serum total bilirubin (mg/dl), and serum AST (U/L). A Cox proportional hazard model used these metrics to predict survival through the formula:

$$\ln\left\{\frac{\text{h}\left(\text{t}\right)}{{\text{h}}_{0}\left(\text{t}\right)}\right\}=1.043*\text{proportion}\;\text{of}\;\text{ducts}\;\text{with}\;\text{diameter}\;3-5\;\text{mm}+3.354*\text{total}\;\text{bilirubin}+2.147*\text{AST}$$

A unit increase in the proportion of bile ducts with diameter 3–5 mm, total bilirubin, and AST significantly increased the hazard ratio of an event by 1.043 (95% CI: 1.003, 1.085), 3.354 (95% CI: 2.125, 5.294) and 2.147 (95% CI: 1.082, 4.263) respectively. Creating an M+BA risk score using the model coefficients was found to significantly increase the hazard ratio of an event by 2.72 (95% CI: 1.97, 3.75) (Fig. 3).

**Fig. 4:**
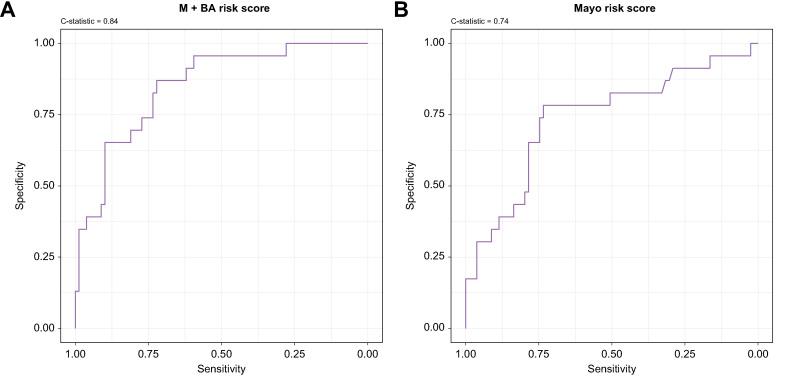
Receiver operator curves. Diagnostic accuracy of (A) the M+BA risk score and (B) the Mayo risk score of discriminating death or liver transplant in the training cohort.

The M+BA risk score had excellent discrimination of transplant-free survival in the training and validation datasets with Harrell’s C-statistic of 0.84 (95% CI: 0.75, 0.93; Fig. 4) and 0.86 (95% CI: 0.77, 0.95) respectively. Using Youden’s index for the training dataset, a threshold for classifying high-risk individuals using the M+BA risk score was established as those with an M+BA risk score greater than 4.41.

**Fig. 5:**
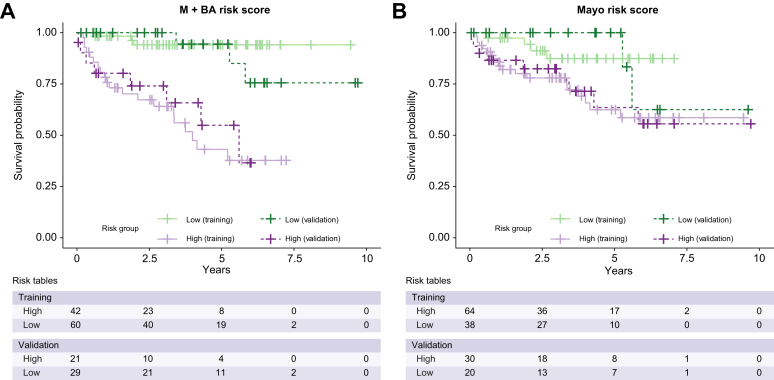
Kaplan–Meier curves. Survival curves for individuals classified as high risk and low risk by (A) the M+BA risk score and (B) the Mayo risk score in both the training and validation data sets.

Applying this threshold, participants classified as high risk by the M+BA risk score had a significantly higher hazard ratio of experiencing death or liver transplant in the validation cohort (5.76 [95% CI: 1.50, 22.07], *p* = 0.01) (Fig. 5). Participants classified as high risk by the Mayo risk score had non-significant hazard ratios of 3.13 (95% CI: 0.67, 14.62, *p* = 0.15) using the previously published threshold. The M+BA risk score was a better fit for explaining transplant-free survival with an AIC of 66.40 compared with the AIC of 71.38 for the Mayo risk score. In the validation cohort, the M+BA risk score outperformed the Mayo risk score in correctly classifying PSC patients who experienced transplant and/or death in ≤3 years. For >3 years, the M+BA risk score outperformed the Mayo risk score in predicting transplant-free survival in patients with PSC, but was slightly worse at correctly identifying a clinical event (Table 2).

**Table 2:** Sensitivity, specificity, positive predictive value (PPV) and negative predictive value (NPV) of the M+BA risk score and Mayo risk score when categorising individuals on likelihood of liver transplant/death in the short term (≤3 years) and long term (>3 years).

Years	n	Risk score	Sensitivity	Specificity	PPV	NPV
≤3 years	23	M+BA	1	0.61	0.42	1
		Mayo	1	0.44	0.33	1
>3 years	27	M+BA	0.50	0.71	0.27	0.83
		Mayo	0.67	0.48	0.33	0.83

The exploratory analysis, in which training and validation datasets were pooled returned an extended version of the M+BA risk score, which contained proportion of ducts with diameter of 3–5 mm, maximum absolute severity of dilation, total stricture score, serum total bilirubin, ALP, creatinine, and ALT. This extended risk score also demonstrated excellent discrimination of transplant-free survival (Harrell’s C-statistic of 0.86 [95% CI: 0.78, 0.93]). However, adding additional metrics did not provide a significant improvement above the M+BA risk score in this cohort (see the Supplementary material for full details).

## Discussion

The present study is the first to derive and validate a prognostic model using quantitative MRCP. We evaluated 152 participants for up to 10 years (median follow-up of 3 years), during which 34 of the 152 participants experienced adverse liver-related outcomes (18 liver transplants, 16 deaths). We utilised quantitative MRCP (MRCP+) to derive a prognostic score with high accuracy and then validated the risk score in a separate cohort of patients with PSC. Using data-driven techniques, three predictive metrics were identified including one MRCP+ metric (the proportion of the bile ducts with diameter 3–5 mm) and two serum biochemical markers (AST and total bilirubin). When combined to create a risk score, the M+BA risk score predicted survival with excellent discriminative performance (AUROC = 0.86).

These results highlight the value of MRCP+ as a tool that may support biochemical investigations of this severe and poorly understood disease.

Using quantitatively derived metrics, MRCP+ overcomes the challenges associated with the significant inter-reader variability and subjective interpretation of standard MRCP images, which have thus far frustrated imaging-derived biomarkers for PSC.^18^ To date, the clinical risk scores applied in PSC, such as the Mayo risk score, rely predominantly on indirect assessments of biliary health such as blood biomarkers and age. These factors have proven effective at predicting short-term survival in end-stage disease. However, given that biochemical changes only occur in later stages of the disease, the power to discriminate early stages or predict progression before end-stage disease has been limited, as evidenced in this study by the low specificity observed with longer survival times. By directly assessing the biliary tree and reducing the dependence on indirect blood-based biomarkers, the M+BA biomarker correctly identified patients at risk of adverse clinical events.

Given the heterogeneous nature of PSC and its long time course, we propose that the M+BA risk score could form part of a panel of relevant markers, including blood biomarkers, which taken together capture different aspects of the disease. For example, there are emerging technologies that measure liver stiffness using ultrasound-based vibration-controlled transient elastography (VCTE) or magnetic resonance elastography (MRE). Some investigators have assessed the role of measuring liver stiffness in patients with PSC. Both baseline liver stiffness measurement (LSM) and change over time seem to be predictive of liver-related outcomes.^9^^,^^10^^,^^13^^,^^32^ These studies report a significant discriminatory ability of LSM values to identify PSC patients with advanced liver fibrosis.^10^^,^^13^^,^^32^ However, studies correlating these measures to clinical outcomes are few, and LSM may be falsely increased at times of increased biliary pressure from choledocholithiasis and cholangitis.^33^ Therefore, the proposed M+BA risk score may have a complementary role with MRE or VCTE.

Quantitative MRCP produces a potential suite of 92 metrics. Reducing the number of features of interest to an interpretable number is, therefore, key. This study used data-driven techniques to select the most appropriate metrics. Reassuringly, the measures identified by the stepwise regression process are in excellent agreement with typically clinically recognised features of PSC. For instance, the impact of dilations and ductal wall thickening is captured through the chosen metric of duct diameter. This metric includes proportion of the biliary tree with diameter ranging from 3–5 mm, which is often observed in cases with multi-focal fibrotic strictures, dilations, and reduced branching of the biliary tree. These findings echo previously reported observations of obliteration of the small peripheral ducts, which generates the ‘pruned-tree’, in patients with more advanced PSC.^34^

Future studies are now needed to further test the derived M+BA risk score in an additional larger cohort, to assess its impact on clinical decision-making and patient outcomes, and to examine how changes to the parameters over time relate to clinical outcomes. Sample size remains a limiting factor when investigating a rare disease over a long-time course. However, the ability to derive an M+BA risk score from retrospective data should help to overcome some of these issues, as many patients with PSC already have long-term MRCP follow-up data. The quantitative and semi-automatic nature of quantitative MRCP will enable the longitudinal progression of these metrics to be monitored and linked, using robust statistical methods, to eventual outcomes. This will help to address a key limitation of risk scores calculated from a baseline assessment, that they fail to account for changes to parameters over time. Although in this study we were able to demonstrate the performance of the M+BA risk score’s predictive potential in both the short (≤3 years) and longer (>3 years), future longitudinal studies, ideally following patients up annually until an outcome is reached, should examine whether changes in quantitative MRCP metrics over time continue to predict outcomes. Given that, in many centers, MRCP data are collected annually, and quantitative MRCP analysis can be generated from most 3D MRCP datasets, patients would not be required to undergo additional investigations, thus facilitating this type of longitudinal study. Typically, such changes over time would be assessed by eye; however, the repeatable nature of MRCP+ technology will allow for quantitative assessment of any changes, better informing prognosis at the individual patient level.

In our exploratory analysis, we identified a second model which, derived from the cohort as a whole, included an expanded number of metrics: the proportion of bile ducts with diameter 3–5 mm, the stricture score sum, the absolute maximum diameter of dilatation severity, creatinine, total bilirubin, ALT, and ALK. This second model also demonstrated excellent discriminative performance. Although the lack of a validation cohort is an important caveat for this second expanded model, the additional power of a larger sample size suggests there may be further predictive signal provided by quantitative MRCP metrics to uncover.

A larger population of patients would also address limitations regarding the representativeness of the population.^35^ The relatively high number of clinical events over the study period suggests that this population may be one of advanced disease referred to a specialist tertiary center. Future studies could examine then validate the prognostic ability of the M+BA biomarker at different stages of the disease.

Once incorporated into clinical assessment, quantitative assessment of the biliary tree may facilitate the development and evaluation of novel treatment options, which are sorely needed to improve the prognosis of PSC patients. Although MRCP is the current gold-standard assessment tool for PSC, the variable and qualitative nature of the clinical information obtained from traditional MRCP scans makes it difficult to incorporate standard MRCP results into clinical risk scores. In this study, we have demonstrated the excellent sensitivity and specificity of quantitative MRCP metrics utilised within a clinical risk index and we have also shown the additional discriminatory power that the wide range of information contained in an MRCP image can bring when assessing long-term patient outcomes. Indeed, by collapsing the quantitative MRCP metrics to an easily interpretable risk score, while also accounting for traditional biochemical assessments, it may be possible to identify patients for whom more frequent follow up may be beneficial and reassure patients with quiescent disease. Such an outcome would ensure that patients receive the most appropriate care in a timely fashion and facilitate more efficient utilisation of healthcare resources. This is particularly true given that MRCP+ can be applied to already collected 3D MRCP data, allowing the M+BA risk score to be calculated retrospectively.

### Conclusions

We generated an objective composite prognostic biomarker, M+BA risk score, that predicts liver transplant or death with greater accuracy than the Mayo risk score. We validated the M+BA risk score in an independent cohort and demonstrated that the predictive power remained high. In doing so, we have shown good evidence for MRCP+ as a novel technology in predicting outcomes for PSC.

## Financial support

No external funding was provided for the conduct of this study.

## Conflicts of interest

RAT, LY, SM, and CF are employed by Perspectum Ltd. The other authors declare no conflicts of interests.

Please refer to the accompanying ICMJE disclosure forms for further details.

## Authors’ contributions

Contributed to study concept, design, data-analysis: RV, VA, CK, MG, FA, NC. Statistical design and analysis: AT, SM, LY. Contributed to MRCP+ analysis: CF. Contributed to writing: RV, VA, CK, MG, FA, NC, AT, CF, LY. Contributed to editing: all authors. Approval of the resulting manuscript: all authors.

## Data availability statement

The data that support the findings of this study are available on request from the corresponding author (RV). The data are not publicly available owing to privacy restrictions.
