# Supplementary material for: A composite score using quantitative magnetic resonance cholangiopancreatography predicts clinical outcomes in primary sclerosing cholangitis
Source: JHEP Rep. 2023 Jun 29;5(10):100834. doi: 10.1016/j.jhepr.2023.100834 (PMC10472223; doi:10.1016/j.jhepr.2023.100834)
Supplement: Multimedia component 1 [file mmc1.pdf]

Supplementary materials

A composite score using quantitative magnetic resonance cholangiopancreatography predicts clinical outcomes in primary sclerosing cholangitis

Raj Vuppalanchi, Vijay Are, Alison Telford, Liam Young, Sofia Mouchti, Carlos Ferreira, Carla Kettler, Mark Gromski, Fatih Akisik, Naga Chalasani

Table of contents

Supplementary results 2

Derivation of an extended M+B Risk score 2

Diagnostic performance of extended M+B Risk score 2

Fig. S1. Forest plot of extended M+B Risk score metrics 3

Fig. S2. Receiver operator curve of extended M+B Risk score 4

Fig. S3. Kaplan-Meier Curve of extended M+B Risk score 5

Table S1. Summary statistics of quantitative MRCP and biochemical metrics for patients who survived transplant-free or had a clinical event. 6

Table S2. Hazard ratios of a unit increase in the quantitative MRCP and blood metrics chosen by the Stepwise Cox regression model. 9

Supplementary CTAT Table 9

Software 9

Details of the corresponding methods author for the manuscript 9

Supplementary results

Derivation of an extended M+B Risk score

A secondary analysis was performed using the same statistical methodology on a pooled cohort consisting of both the test and validation cohorts. The stepwise regression method for Cox's Proportional Hazard model returned the Risk Score of quantitative MRCP and blood metrics (extended M+B Risk Score). In the larger cohort, seven metrics were included in the extended M+B model (Fig. S1) including: proportion of ducts with diameter 3-5 mm (%), the total stricture score, the maximum absolute severity of dilation (mm), serum total bilirubin (mg/dL), serum alkaline phosphatase (iU), serum ALT (iU), and serum creatinine (mg/dL). A Cox proportional hazard model used these metrics to predict survival through the formula:

$$\ln\left\{\frac{\mathrm{h(t)}}{\mathrm{h_0(t)}}\right\} = 1.065 \times \text{Proportion of the biliary tree with diameter 3-5mm } +$$

$$\begin{aligned}
&1.232 \times \text{Maximum absolute severity of dilations} + \\
&0.957 \times \text{Total stricture score} + \\
&3.571 \times \text{Total Bilirubin } + \\
&4.842 \times \text{ Creatinine} + \\
&1.129 \times \text{ALK} + \\
&0.446 \times \text{ ALT}
\end{aligned}$$

A unit increase in each of the metrics significantly increased the hazard ration of a clinical event for each metric. The changes in hazard ratio for a unit increase are shown in Table S2. Creating a composite extended M+B Risk Score using the model coefficients was found to significantly increase the hazard ratio of an event by tbc (Fig. S2).

Diagnostic performance of extended M+B Risk score

The extended M+B Risk Score had excellent discrimination of transplant-free survival with Harrell's C-statistic of 0.86 (95% CI: 0.78, 0.93; Fig. S3). The extended M+B score had marginally higher diagnostic performance than the M+BA score derived on a subset of the data. However, this was not significant and the M+BA score maintained excellent diagnostic performance (C-statistic 0.84 [95% CI: 0.77 – 0.91]). Using the Youden's index for the training dataset, a threshold for classifying high-risk individuals using the extended M+B Risk Score was established as those with an extended M+B Risk score of greater than 4.63.

Applying this threshold, participants classified as high-risk by the extended M+B Risk Score had significantly higher hazard ratio of experiencing death or liver transplant (11.66 [95% CI: 5.04, 26.97]) (Fig. S4). Participants classified as high-risk by the Mayo risk score had hazard ratios of 3.27 (95% CI: 1.35, 7.91) using the previously published threshold. The extended M+B Risk Score was found to be a better fit for explaining transplant-free survival with a AIC of 266.67 compared to the AIC of 301.68 for the Mayo Risk Score.

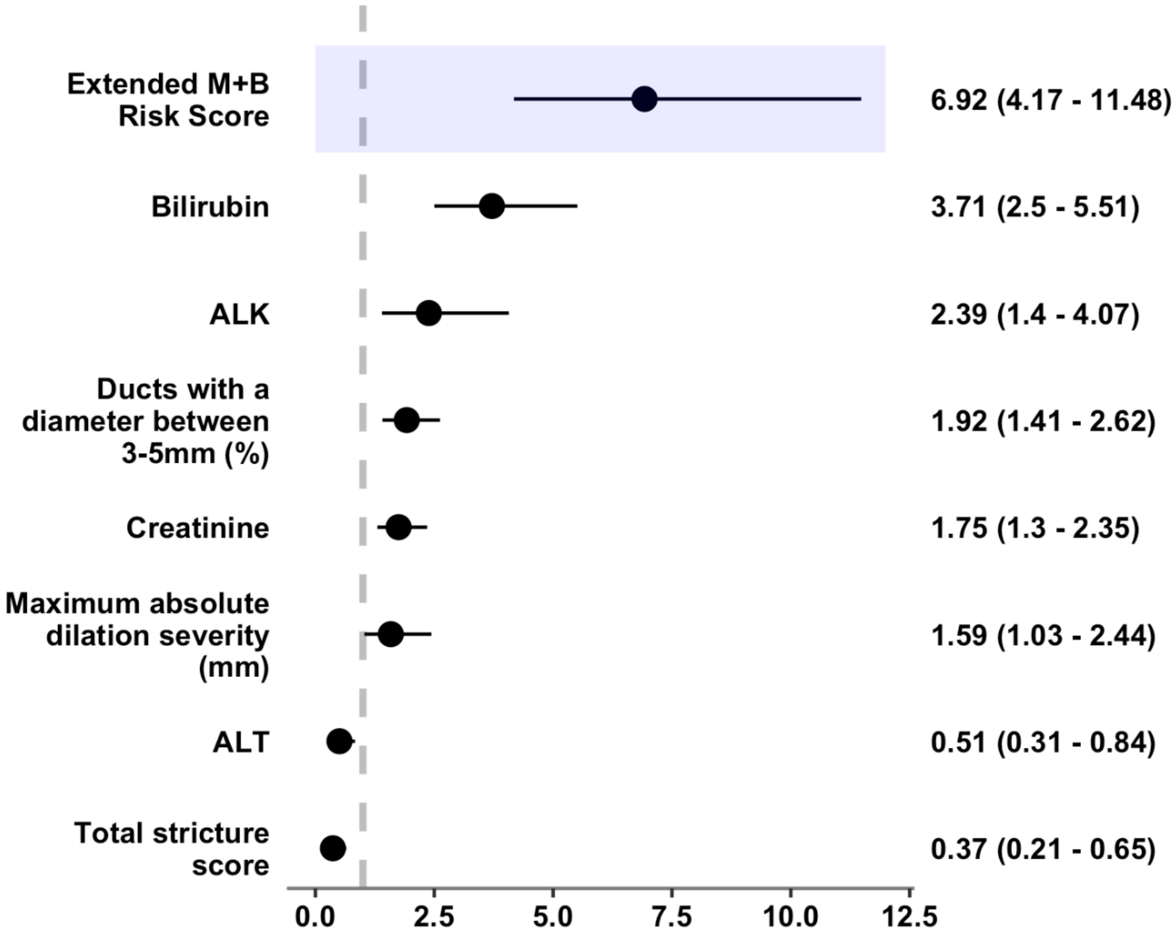

Fig. S1: Forest plot of extended M+B Risk score metrics. Shaded box: Hazard Ratio and corresponding 95% confidence interval of the extended M+B Risk score. Below the shaded box: Hazard Ratios and corresponding 95% confidence interval of the quantitative MRCP and blood metrics chosen by the Stepwise Cox regression model. All the metrics were standardised as z-scores.

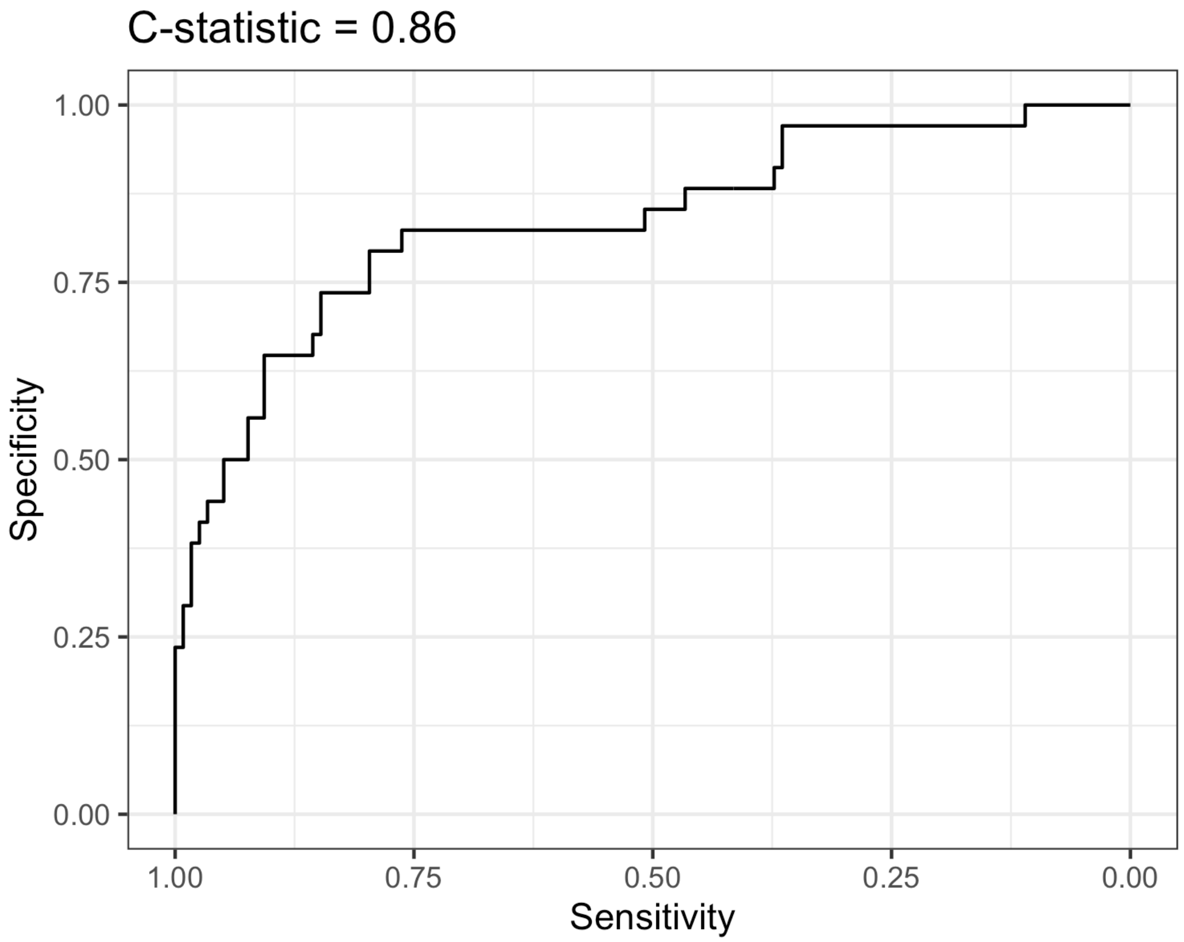

Fig. S2: Receiver operator curve of extended M+B Risk score. Diagnostic accuracy of the extended M+B Risk of discriminating death or liver transplant in the combined cohort.

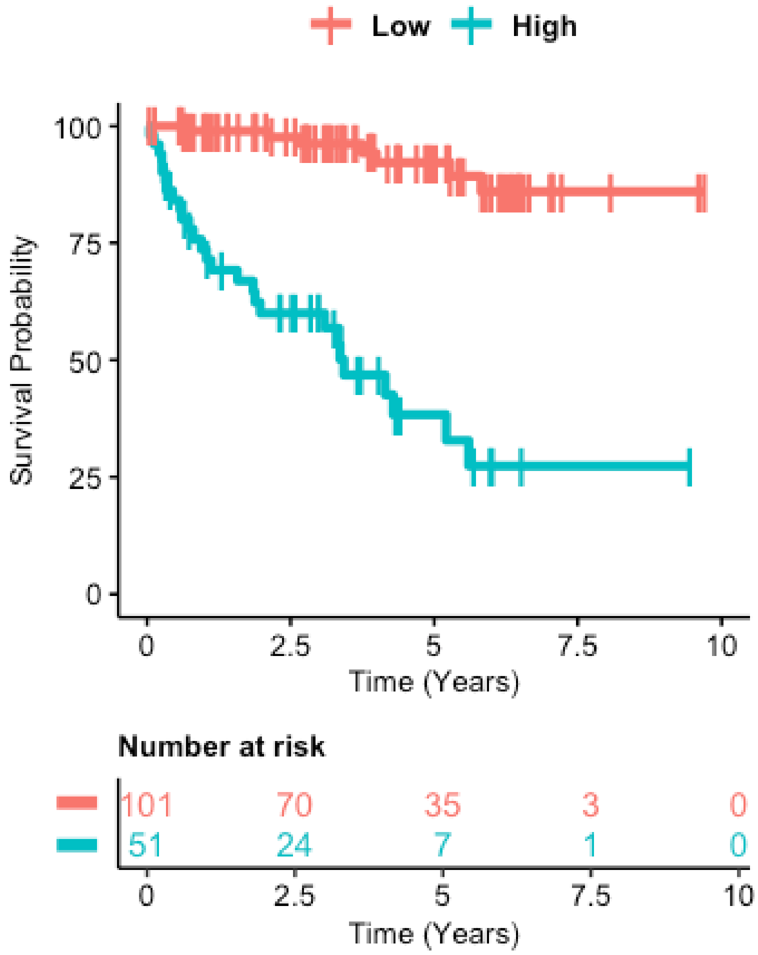

Fig. S3. Kaplan-Meier Curve of extended M+B Risk score. Survival curves for individuals classified as high-risk and low-risk by the extended M+B Risk Score the combined data set.

Table S1. Summary statistics of quantitative MRCP and biochemical metrics for patients who survived transplant-free or had a clinical event. Metrics in italics were those that were included in the stepwise model selection. Data are presented as mean (SD) or median (IQR) for continuous variables, and as numbers and percentages (%) for categorical data.

Metric	All	No Liver Transplant/ Death	Liver Transplant/ Death	p-value*
**Quantitative MRCP**				
Number of ducts (n)	64 (33, 102)	66 (35, 101)	52 (21, 102)	0.6
Number of strictures (n)	7 (3, 12)	7 (3, 14)	8 (2, 12)	0.7
Number of dilations (n)	14 (6, 25)	14 (6, 26)	16 (5, 23)	0.7
Number of strictures with a length < 3 mm (n)				0.7
0	98 (64%)	79 (67%)	19 (56%)	
1	38 (25%)	26 (22%)	12 (35%)	
2	12 (7.9%)	10 (8.5%)	2 (5.9%)	
3	3 (2%)	2 (1.7%)	1 (2.9%)	
6	1 (0.7%)	1 (0.8%)	0 (0%)	
Number of strictures with a length between 3-10 mm	5.0 (2.0, 9.2)	5.0 (2.0, 10.0)	5.0 (1.3, 9.0)	0.7
Number of ducts containing one or more stricture (n)	6.0 (3.0, 10.0)	6.0 (3.0, 10.0)	6.0 (2.0, 10.0)	0.7
Number of ducts containing one or more dilation (n)	12 (5, 19)	12 (5, 20)	12 (4, 18)	0.7
Number of ducts containing one or more stricture and dilation (n)	4.0 (1.0, 7.0)	4.0 (1.0, 7.0)	4.0 (1.0, 6.0)	0.7
Number of ducts containing one or more stricture OR dilation (n)	14 (6, 22)	14 (8, 23)	14 (5, 22)	0.7
Total length of ducts (mm)	1059 (549, 1912)	1093 (568, 2011)	829 (329, 1778)	0.3
Total length of strictures (mm)	52 (21, 95)	52 (23, 97)	52 (16, 88)	0.6
Total length of dilations (mm)	86 (36, 163)	85 (38, 161)	87 (28, 160)	0.7
Total length of strictures and dilations (mm)	150 (58, 262)	152 (61, 259)	143 (37, 256)	0.7

Proportion of the biliary tree with diameter between 5-7 mm (%)	0.029 (0.013, 0.048)	0.029 (0.012, 0.045)	0.030 (0.017, 0.057)	0.3
Proportion of the biliary tree with diameter between 7-9 mm (%)	0.004 (0.000, 0.012)	0.003 (0.000, 0.011)	0.008 (0.003, 0.018)	0.2
Proportion of bile ducts with a diameter between 3-5 mm (%)	19 (13, 28)	18 (12, 27)	24 (16, 33)	0.029
Proportion of bile ducts with a diameter between 5-7 mm (%)	1.53 (0.00, 4.00)	1.47 (0.00, 3.81)	1.62 (0.00, 4.59)	0.7
Maximum length of ducts (mm)	80 (67, 100)	82 (67, 100)	77 (59, 92)	0.7
Maximum absolute severity of strictures (mm)	2.24 (1.71, 3.33)	2.23 (1.70, 3.34)	2.28 (1.79, 3.20)	0.9
Maximum absolute severity of dilatations (mm)	3.82 (2.54, 5.11)	3.59 (2.56, 5.04)	4.18 (2.58, 5.83)	0.7
Maximum relative severity of dilatations (%)	1.30 (0.93, 1.88)	1.28 (0.93, 1.80)	1.42 (0.99, 2.00)	0.7
Sum of the severity score for strictures	23 (9, 41)	23 (10, 42)	21 (6, 28)	0.6
Sum of severity scores for dilatations	58 (24, 109)	57 (26, 109)	66 (18, 104)	0.9
Sum of absolute severity of strictures (mm)	11 (4, 21)	11 (5, 21)	13 (3, 20)	0.7
Sum of absolute severity of dilatations	26 (10, 48)	24 (10, 47)	30 (12, 47)	0.9
Sum of relative severity of strictures (%)	3.2 (1.3, 5.5)	3.1 (1.5, 5.5)	3.4 (0.7, 5.4)	0.7
Sum of relative severity of dilatations (%)	9 (4, 17)	9 (4, 18)	11 (3, 16)	0.7
**Biochemical**				
Sodium	4.927 (4.913, 4.934)	4.927 (4.913, 4.934)	4.920 (4.905, 4.927)	0.058
Creatinine	-0.21 (-0.37, -0.02)	-0.21 (-0.36, -0.01)	-0.25 (-0.43, -0.02)	0.7
Albumin	1.34 (1.22, 1.42)	1.36 (1.28, 1.44)	1.18 (1.01, 1.31)	<0.001

Bilirubin	0.10 (-0.36, 0.93)	0.00 (-0.51, 0.41)	1.36 (0.14, 1.98)	<0.001
ALT	4.03 (3.33, 4.54)	3.97 (3.27, 4.54)	4.30 (3.74, 4.53)	0.3
AST	3.95 (3.40, 4.44)	3.87 (3.33, 4.227)	4.30 (4.03, 4.77))	0.001
ALK	5.46 (4.85, 6.08)	5.38 (4.80, 5.93)	6.02 (5.31, 6.26)	0.018
Platelet Count	5.39 (5.06, 5.65)	5.44 (5.16, 5.65)	5.37 (4.92, 5.62)	0.7
INR	1.09 (1.01, 1.23)	1.08 (1.00, 1.18)	1.19 (1.07, 1.42)	0.022
**Other**				
Mayo Risk Score	0.16 (-0.36, 1.16)	0.08 (-0.51, 0.61)	1.37 (0.59, 2.39)	<0.001
Mayo Risk Score Categorical				<0.001
High	16 (11%)	5 (4.2%)	11 (32%)	
Intermediate	78 (51%)	61 (52%)	17 (50%)	
Low	58 (38%)	52 (44%)	6 (18%)	

Table S2. Hazard ratios of a unit increase in the quantitative MRCP and blood metrics chosen by the Stepwise Cox regression model. All blood metrics are log transformed prior to assessment.

Metric*	HR (95% CI)
Proportion of ducts with diameter 3-5 mm	1.06 (1.03 – 1.10)
Maximum absolute severity of dilation	1.23 (1.01 – 1.50)
Total stricture score	0.96 (0.93 – 0.98)
serum total bilirubin	3.57 (2.43 – 5.24)
ALK	3.09 (1.55 – 6.18)
Creatinine	4.84 (2.11 – 11.13)
ALT	0.45 (0.25 – 0.81)

Supplementary CTAT Table

Software

Software name	Manufacturer	Version
**MRCP++**	**Perspectum Ltd, Oxford, UK**	**V1.0.0**

Details of the corresponding methods author for the manuscript

Raj Vuppalanchi, MD
Professor of Medicine,
Division of Gastroenterology and Hepatology,
Indiana University School of Medicine,
702 Rotary Circle, Suite 225,
Indianapolis, IN 46202, USA
rvuppala@iu.edu
