# Supplementary material for: A composite score using quantitative magnetic resonance cholangiopancreatography predicts clinical outcomes in primary sclerosing cholangitis
Source: JHEP Rep. 2023 Jun 29;5(10):100834. doi: 10.1016/j.jhepr.2023.100834 (PMC10472223; doi:10.1016/j.jhepr.2023.100834)
Supplement: Multimedia component 4 [file mmc4.pdf]

Research article

JHEP|Reports

A composite score using quantitative magnetic resonance cholangiopancreatography predicts clinical outcomes in primary sclerosing cholangitis

Raj Vuppalanchi,[1,*] Vijay Are,[1] Alison Telford,[2] Liam Young,[2] Sofia Mouchti,[2] Carlos Ferreira,[2] Carla Kettler,[1] Mark Gromski,[1] Fatih Akisik,[3] Naga Chalasani[1]

[1]*Division of Gastroenterology and Hepatology, Indiana University School of Medicine, Indianapolis, IN, USA;* [2]*Perspectum Ltd., Oxford, UK;* [3]*Department of Radiology, Indiana University School of Medicine, Indianapolis, IN, USA*

JHEP Reports **2023.** https://doi.org/10.1016/j.jhepr.2023.100834

Background & Aims: Magnetic resonance cholangiopancreatography (MRCP) for evaluation of biliary disease currently relies on subjective assessment with limited prognostic value because of the lack of quantitative metrics. Artificial intelligence-enabled quantitative MRCP (MRCP+) is a novel technique that segments biliary anatomy and provides quantitative biliary tree metrics. This study investigated the utility of MRCP+ as a prognostic tool for the prediction of clinical outcomes in primary sclerosing cholangitis (PSC).
Methods: MRCP images of patients with PSC were post-processed using MRCP+ software. The duration between the MRCP and clinical event (liver transplantation or death) was calculated. Survival analysis and stepwise Cox regression were performed to investigate the optimal combination of MRCP+ metrics for the prediction of clinical outcomes. The resulting risk score was validated in a separate validation cohort and compared with an existing prognostic score (Mayo risk score).
Results: In this retrospective study, 102 patients were included in a training cohort and a separate 50 patients formed a validation cohort. Between the two cohorts, 34 patients developed clinical outcomes over a median duration of 3 years (23 liver transplantations and 11 deaths). The proportion of bile ducts with diameter 3–5 mm, total bilirubin, and aspartate aminotransferase were independently associated with transplant-free survival. Combined as a risk score, the overall discriminative performance of the MRCP+ risk score (M+BA) was excellent; area under the receiver operator curve 0.86 (95% CI: 0.77, 0.95) at predicting clinical outcomes in the validation cohort with a hazard ratio 5.8 (95% CI: 1.5, 22.1). This was superior to the Mayo risk score.
Conclusions: A composite score combining MRCP+ with total bilirubin and aspartate aminotransferase (M+BA) identified PSC patients at high risk of liver transplantation or death. Prospective studies are warranted to evaluate the clinical utility of this novel prognostic tool.
Impact and Implications: Primary sclerosis cholangitis (PSC) is a disease of the biliary tree where inflammation and fibrosis cause areas of narrowing (strictures) and expansion (dilatations) within the biliary ducts leading to liver failure and/or cancer (cholangiocarcinoma). In this study, we demonstrate that quantitative assessment of the biliary tree can better identify patients with PSC who are at high risk of either death or liver transplantation than a current blood-based risk score (Mayo risk score).
© 2023 The Authors. Published by Elsevier B.V. on behalf of European Association for the Study of the Liver (EASL). This is an open access article under the CC BY license (http://creativecommons.org/licenses/by/4.0/).

Introduction

Primary sclerosing cholangitis (PSC) is a chronic and progressive cholestatic liver disease characterised by multi-focal stricturing throughout the biliary tree.[1–4] Currently, liver transplantation is the only lifesaving intervention. Although there are no effective pharmacotherapies approved, accurately characterising the disease course is of critical importance for developing new, effective, and affordable treatments. Identifying those at highest risk of poor outcomes is an integral part of the clinical assessment to stratify those at the most need of intervention with endoscopic retrograde cholangiopancreatography (ERCP) and, or referral to liver transplantation in a timely manner.[3,4] Opportunities for intervention are made more challenging by the rareness of symptoms in early-stage PSC alongside a lack of effective biomarkers.[3–5] Although several risk scores and biomarkers have been developed to estimate rates of disease progression,[6–12] their performance remains limited[13] and relies heavily upon biochemical markers which may fluctuate or lag behind parenchymal and ductal changes.[14]

Current clinical guidelines for the diagnosis and management of PSC are based on the cholangiographic or histological features of sclerosing cholangitis in the absence of identifiable causes of

Keywords: MRCP; Non-invasive; Quantitative MRI; PSC; Prognostic biomarkers.
Received 19 August 2022; received in revised form 14 February 2023; accepted 16 June 2023; available online 29 June 2023
* Corresponding author. Address: Division of Gastroenterology and Hepatology, Indiana University School of Medicine, 702 Rotary Circle, Suite 225, Indianapolis, IN 46202 USA. Tel.: +1-317-278-1652; Fax: +1-317-278-1949.
E-mail address: rvuppala@iu.edu (R. Vuppalanchi).

ELSEVIER

EASL The Home of Hepatology

secondary sclerosing cholangitis.[3,4] Although ERCP is the traditional gold standard, there is an associated risk of pancreatitis or cholangitis.[3,4] Given that around 60% of patients with large duct PSC show evidence of radiological progression over a 4-year follow up,[15] non-invasive imaging-based investigations such as magnetic resonance cholangiopancreatography (MRCP) are now recommended in patients who have new or changing symptoms, or evolving abnormalities in laboratory investigations.[3,4,16] Despite the widespread use of MRCP, there remain important limitations, including poor bile duct depiction as well as subjective assessments and unstandardised acquisition protocols. Recently, artificial intelligence-enabled (AI-enabled) software has been developed that enhances conventional MRCP to produce quantitative MRCP (MRCP+™, Perspectum Ltd, Oxford, UK). The software produces a 3D model of the biliary tree which aids visualisation, but more importantly, provides novel quantitative measures for the direct assessment of ductal anatomy.[17] Because of the heterogeneity of bile duct alterations observed in PSC, the adoption of quantitative evaluation of MRCP images has the potential to improve diagnostic performance, reduce clinician burden and sensitively monitor ductal change over time.[18] MRCP+ calculates quantitative 3D biliary system models from historical MRCP images, enabling the measurement of bile duct widths and automatic detection of regions of variation of duct widths. It also allows for regional volumetric analysis of the biliary tree, pancreatic duct, and gallbladder (Fig. 1).

In recent studies, multiple MRCP+ parameters were closely associated with biochemical scoring systems and magnetic resonance elastography (MRE) and demonstrated the potential to act as a risk stratification tool in PSC.[17,19] Until recently, it was unknown how to combine multiple predictive metrics with other measures to provide a clear probability of an outcome. This approach of combining a panel of relevant markers, which individually capture aspects of disease may prove useful in providing a more granular assessment. However, as highlighted by Moons *et al.*[20] deriving a prognostic model is challenging, requiring multiple studies to first develop the new score and assess its promise using internal validation, then validate the new score in external cohorts, before finally assessing the impact of the prognostic model on clinical practice.[20–22] Two recent studies have now taken the first steps of developing a prognostic model. Cristoferi *et al.*[23] and Cazzagon *et al.*[24] independently developed prognostic models that contained quantitative MRCP metrics. In their study of 87 patients with PSC Cristoferi *et al.*[23] found that combining the count of number of strictures with a measurement of spleen size enabled the discrimination of survival. Meanwhile, Cazzagon *et al.*[24] found in their study of 77 patients with PSC that median duct diameter was significantly associated with patient outcomes.

Building on these encouraging studies, this new work aimed to both develop and validate a prognostic model, using MRCP+, to predict clinical outcomes, including death and liver transplant, in patients with PSC. We then aimed to compare this new model to the revised Mayo risk score.[12] In doing so, we evaluate the promise of a risk classifier based on a data-driven combination of MRCP+ and blood tests to predict death or liver transplant among patients with PSC.

Patients and methods

Design and study participants

This retrospective study consisted of data initially collected after informed consent as part of prospective databases of PSC patients undergoing ERCP and those attending outpatient hepatology clinics, along with retrospective liver transplant registry data obtained through Organ Transplant Tracking Record Systems at Indiana University Academic Health Center, Indianapolis, IN, USA. Study participants included in the current analysis were those who underwent MRCP and standard biochemical tests required to calculate the revised Mayo risk score.[12] The baseline visits of individuals in this study occurred between June 2009 and August 2019. All participants were followed up for liver transplant and

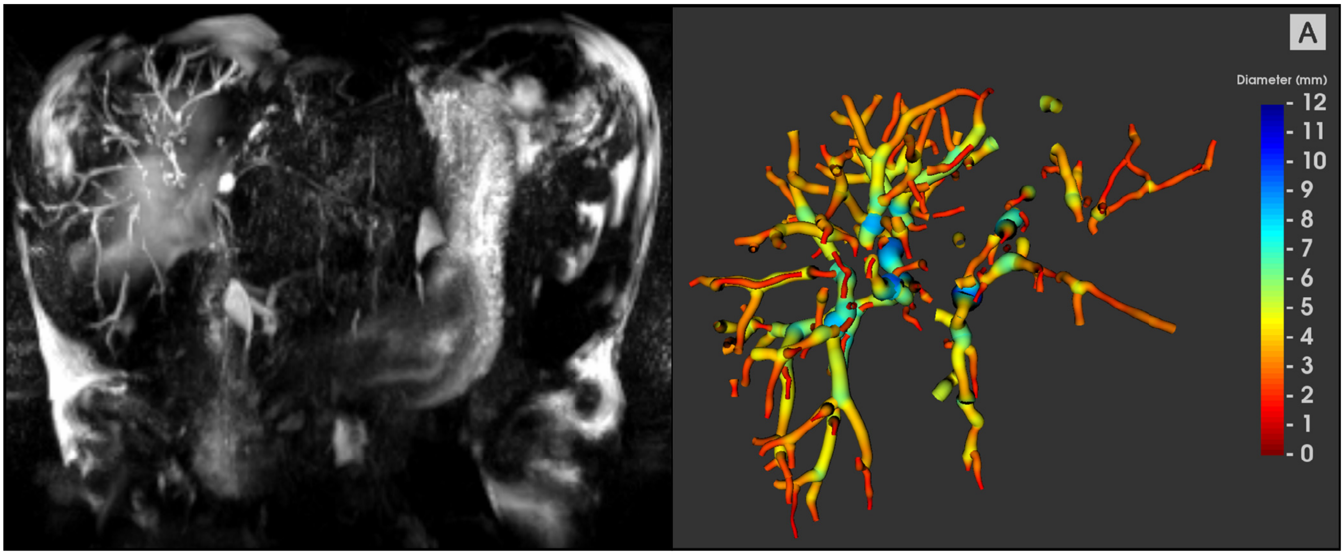

Fig. 1. Example MRCP maximum intensity projection and MRCP+ model from a patient with large duct PSC. In the MRCP+ model, shown on the right, the diameter of each duct is shown by the colourmap and hence regions of dilatation can be clearly seen (blue). Quantitative analyses show a total biliary tree volume of 29.2 ml, 133 modelled ducts, 16 potential strictures, 50 potential dilations, 2538.7 mm of total length of ducts with a 438.7 mm of total length of strictures and dilatations. MRCP, magnetic resonance cholangiopancreatography.

death. This study was approved by the Institutional Review Board at Indiana University School of Medicine. For the current study, patients who had either incomplete MRCP+ metrics or blood biochemistry were excluded. Death date was confirmed through electronic health records or the national death index.[25]

Imaging acquisition

Scans were performed on 1.5 Tesla (T) and 3T clinical magnetic resonance imaging (MRI) scanners available as part of participants' routine clinical evaluation. Heavily T2-weighted MRCP images were acquired using 3D multi-shot fast/turbo spin echo acquisitions with long echo train lengths and short echo spacing to generate 3D volumetric images. Parameters used include an echo time (TE) range from 347 ms to 866 ms and 40–160 contiguous slices with 1–1.2 mm slice thickness. The pixel resolution of the acquired data varied from 0.4 mm × 0.4 mm to 1.2 mm × 1.2 mm. 3D MRCP images were acquired in a coronal oblique orientation to cover the largest extent of the biliary tree and pancreatic duct. Respiratory gating with navigator tracking was used for data acquisition. The expiration phase of the breathing cycle was defined as the period for image acquisition, so the repetition time (TR) varied with the breathing rate. Fat suppression techniques were used to suppress signals from fat, and parallel imaging techniques were used to reduce scanning time.

Imaging processing

Archived MRCP images were assigned a unique study identifier after removing protected health information and then uploaded. These files were then processed with MRCP+ software, which processes 3D MRCP acquisitions using AI-driven pathfinding algorithms and tubular enhancement techniques to derive a quantitative parametric model of the biliary tree and pancreatic ducts.[26] Ninety-two quantitative MRCP metrics that characterise biliary tree structure and include measures of volume and duct length, as well as numbers of strictures and dilatations, were calculated from the MRCP+ models. Detailed information regarding MRCP+ is provided within the Supplementary material. The resulting models were assessed for quality by a trained analyst. An example of MRCP+ is shown in Fig. 1.

Statistical analysis

Analyses were performed using R software version 4.0.2 (R Foundation for Statistical Computing, Vienna, Austria). Descriptive statistics are presented for all participants, median with IQR used to describe continuous variables, and frequency and percentage for categorical variables. The distribution of the continuous variables between the presence and absence of clinical outcome groups was compared with the Wilcoxon Sum of Ranks; the Chi-square test was used to compare the distribution of the presence of a categorical variable.

The prognostic model was constructed and validated using a split-sample approach. Briefly, the cohort was randomly split into a training and validation cohort with a ratio of 3:1. Next, the quantitative MRCP metrics were filtered to ensure only metrics with previously reported good or excellent reproducibility (intraclass correlation [ICC] >0.6) were considered for inclusion in the prognostic model.[27] Biochemical metrics, serum aspartate aminotransferase (AST), alanine aminotransferase (ALT), albumin, platelet count, total bilirubin, alkaline phosphatase (ALP), were log-transformed as previously described by de Vries *et al.*[28]

A stepwise variable selection approach was performed on the training dataset with the reduced set of quantitative MRCP and biochemical metrics input to identify the optimal combination of metrics for the prognostic model.[29] A strict variance inflation factor of 2.5 was chosen to reduce the negative impact of multicollinearity on the model.[30] Discrimination of the model in both the training and validation data sets was assessed through Harwell's C-statistic.

Using the derived model, an M+BA risk score was calculated for each patient. Overall diagnostic accuracy of the M+BA risk score was estimated by the area under the receiver operator curve (AUROC), and an optimal cut-off to categorise individuals as high risk and low risk was derived using Youden's index from the training cohort. In line with previous studies, a single threshold of zero was applied to the revised Mayo risk score to categorise individuals as high-risk and low-risk.[12,31]

The categorisations defined by the M+BA risk score and the revised Mayo risk score were compared by considering the hazard ratios for those classified as high risk by both risk scores, respectively. Kaplan–Meier curves were plotted to present the cumulative probability of transplant-free survival since baseline in both the training and validation cohorts. Akaike's Information Criteria (AIC) was used to assess which risk score provided a better fit for explaining transplant-free survival. The prognostic accuracy of each for predicting death and/or liver transplant in patients with PSC in the short term (≤3 years) and long term (>3 years) was also assessed in the validation cohort.

To investigate whether an increased sample size would reveal any additional signal, we conducted a secondary, exploratory analysis in which both training and validation cohorts were pooled, and the analysis (excluding validation steps) were repeated. This secondary analysis is detailed within the Supplementary material.

Results

Records of 173 patients with PSC with multiple clinic visits were available for review. After applying the exclusion criteria, 152 participants with baseline data were included in the analysis, with 102 patients included in the training cohort (63% male; median BMI of 27 kg/m^2; 84% Caucasian) and 50 forming a validation dataset (76% male; median BMI of 26 kg/m^2; 82% Caucasian). No significant differences in demographics were seen between patients in the test and validation cohorts (Table 1). In the training and validation cohorts, 14 and 4 patients had a liver transplant and 9 and 7 patients died respectively with median transplant-free survival of 3 years for both cohorts (Fig. 2). The percentage of ducts with a diameter between 3 and 5 mm, the Mayo risk score, average albumin, bilirubin, AST, ALP, international normalised ratio, and Fibrosis-4 (FIB4), were significantly different between the group of participants who experienced the clinical event and those who did not (Table S1) but were comparable between the training and validation cohorts.

Prediction of outcomes

The stepwise regression method for Cox's Proportional Hazard model returned the risk score of quantitative MRCP and blood metrics (M+BA risk score). The three M+BA model metrics (Fig. 3) were: proportion of bile ducts with diameter 3–5 mm (%), serum

Table 1. Patient characteristics.

	All	Training cohort	Validation cohort	*p* value*
N (%)	152 (100)	102 (100.0)	50 (100.0)	
Age (years); median (IQR)	48 (32–63)	48 (33–61)	51 (30–66)	>0.9
Sex (males); n (%)	102 (67)	64 (63)	38 (76)	0.5
BMI (kg/m^2); median (IQR)	26.6 (23.2–30.0)	27.1 (23.8–31.0)	25.6 (22.3–28.4)	0.4
Ethnicity; n (%)				>0.9
Caucasian	122 (84)	85 (84)	37 (82)	
African-American	21 (14)	14 (14)	7 (16)	
Other	3 (2.1)	2 (2)	1 (2.2)	
Alcohol abuse; n (%)	16 (11)	13 (13)	3 (6.2)	0.7
Cholecystectomy at baseline; n (%)	13 (8.6)	9 (8.8)	4 (8.0)	>0.9
Type of IBD; n (%)				>0.9
Ulcerative colitis	85 (76)	62 (77)	23 (74)	
Crohn's disease	26 (23)	18 (22)	8 (26)	
Indeterminate colitis	1 (0.9)	1 (1.2)	0 (0.0)	
UDCA class; n (%)				>0.9
Non-user	63 (43)	40 (41)	23 (46)	
Regular dose UDCA	76 (51)	52 (53)	24 (48)	
High dose UDCA	9 (6.1)	6 (6.1)	3 (6.0)	

Data are presented as mean (SD) or median (IQR) for continuous variables, and as numbers and percentages (%) for categorical data.
BMI, body mass index; IBD, inflammatory bowel disease; IQR, interquartile range; UDCA, ursodeoxycholic acid.
* Bonferroni corrected *p* value.

total bilirubin (mg/dl), and serum AST (U/L). A Cox proportional hazard model used these metrics to predict survival through the formula:

$$\ln\left\{\frac{h(t)}{h_0(t)}\right\} = 1.043 * \text{proportion of ducts with diameter } 3-5\text{ mm} + 3.354 * \text{total bilirubin} + 2.147 * \text{AST}$$

A unit increase in the proportion of bile ducts with diameter 3–5 mm, total bilirubin, and AST significantly increased the hazard ratio of an event by 1.043 (95% CI: 1.003, 1.085), 3.354 (95% CI: 2.125, 5.294) and 2.147 (95% CI: 1.082, 4.263) respectively. Creating an M+BA risk score using the model coefficients was found to significantly increase the hazard ratio of an event by 2.72 (95% CI: 1.97, 3.75) (Fig. 3).

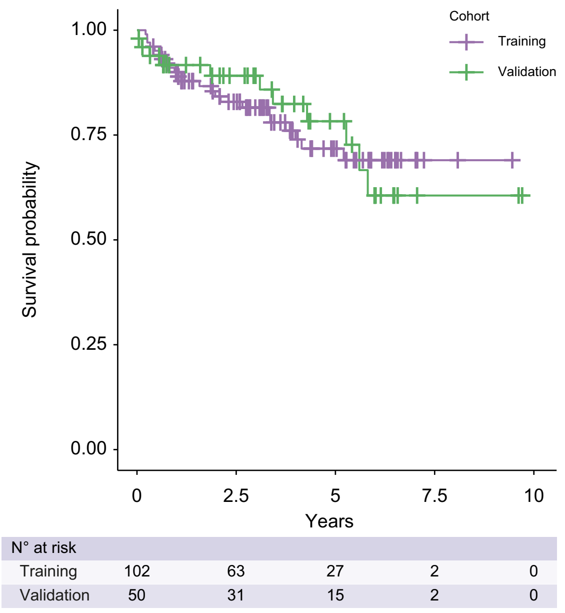

Fig. 2. Kaplan–Meier survival curves for training and validation cohorts.

The M+BA risk score had excellent discrimination of transplant-free survival in the training and validation datasets with Harrell's C-statistic of 0.84 (95% CI: 0.75, 0.93; Fig. 4) and 0.86 (95% CI: 0.77, 0.95) respectively. Using Youden's index for the training dataset, a threshold for classifying high-risk individuals using the M+BA risk score was established as those with an M+BA risk score greater than 4.41.

Applying this threshold, participants classified as high risk by the M+BA risk score had a significantly higher hazard ratio of experiencing death or liver transplant in the validation cohort (5.76 [95% CI: 1.50, 22.07], $p = 0.01$) (Fig. 5). Participants classified as high risk by the Mayo risk score had non-significant hazard ratios of 3.13 (95% CI: 0.67, 14.62, $p = 0.15$) using the previously published threshold. The M+BA risk score was a better fit for explaining transplant-free survival with an AIC of 66.40 compared with the AIC of 71.38 for the Mayo risk score. In the validation cohort, the M+BA risk score outperformed the Mayo risk score in correctly classifying PSC patients who experienced transplant and/or death in ≤3 years. For >3 years, the M+BA risk score outperformed the Mayo risk score in predicting transplant-free survival in patients with PSC, but was slightly worse at correctly identifying a clinical event (Table 2).

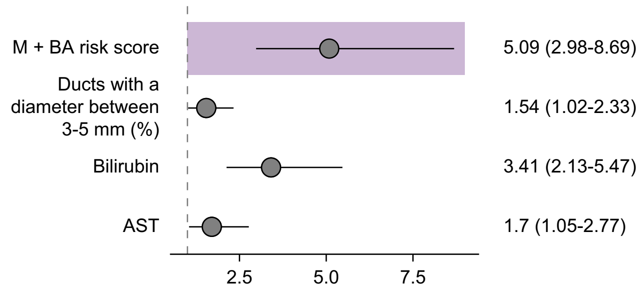

Fig. 3. Forest plot. Shaded box: hazard ratio and corresponding 95% CI of the composite M+BA risk score. Below shaded box: hazard ratios and corresponding 95% CI of the quantitative MRCP and blood metrics chosen by the Stepwise Cox regression model. All metrics were standardised as z-scores. AST, aspartate aminotransferase; MRCP, magnetic resonance cholangiopancreatography.

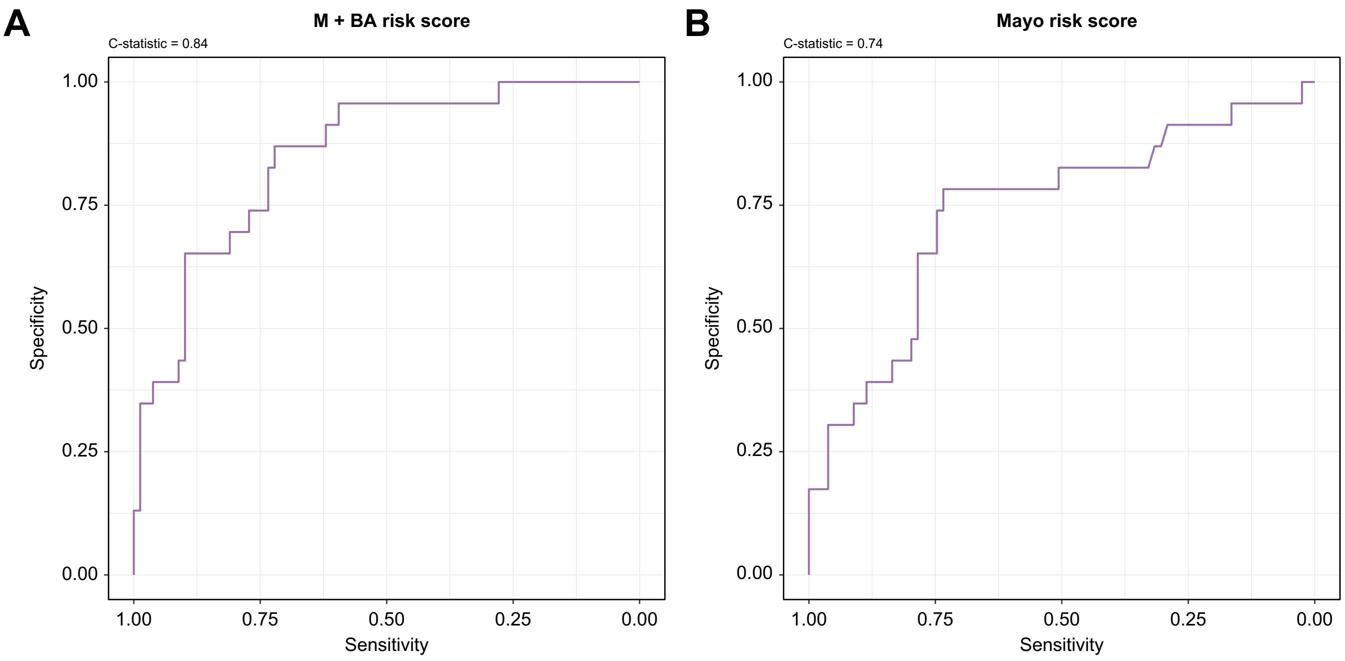

Fig. 4. Receiver operator curves. Diagnostic accuracy of (A) the M+BA risk score and (B) the Mayo risk score of discriminating death or liver transplant in the training cohort.

The exploratory analysis, in which training and validation datasets were pooled returned an extended version of the M+BA risk score, which contained proportion of ducts with diameter of 3–5 mm, maximum absolute severity of dilation, total stricture score, serum total bilirubin, ALP, creatinine, and ALT. This extended risk score also demonstrated excellent discrimination of transplant-free survival (Harrell's C-statistic of 0.86 [95% CI: 0.78, 0.93]). However, adding additional metrics did not provide a significant improvement above the M+BA risk score in this cohort (see the Supplementary material for full details).

Discussion

The present study is the first to derive and validate a prognostic model using quantitative MRCP. We evaluated 152 participants for up to 10 years (median follow-up of 3 years), during which 34

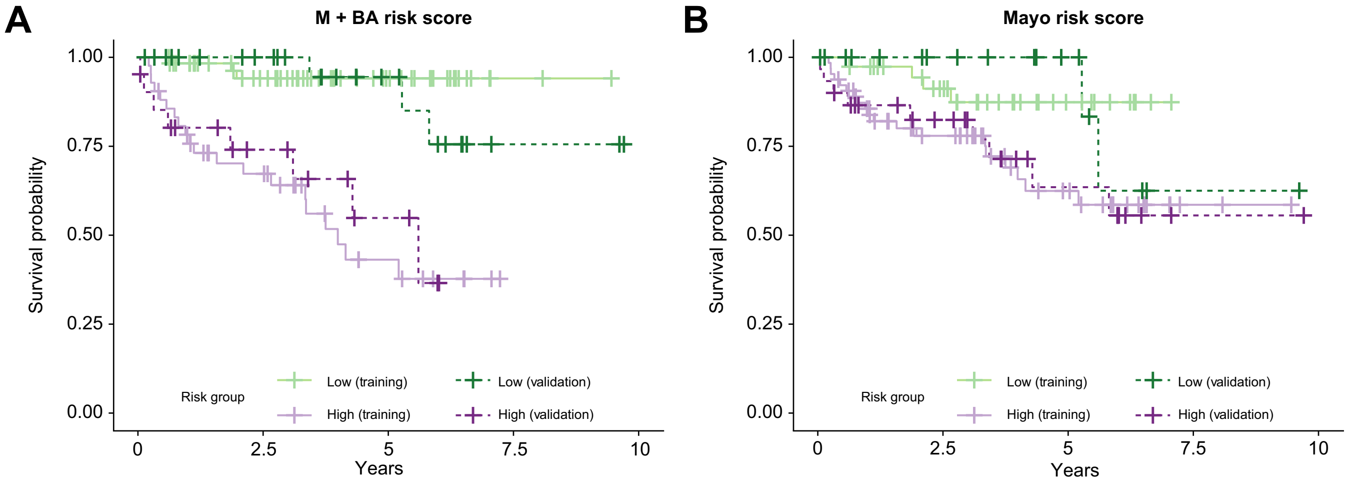

Risk tables (A)

**Training**					
High	42	23	8	0	0
Low	60	40	19	2	0
**Validation**					
High	21	10	4	0	0
Low	29	21	11	2	0

Risk tables (B)

**Training**					
High	64	36	17	2	0
Low	38	27	10	0	0
**Validation**					
High	30	18	8	1	0
Low	20	13	7	1	0

Fig. 5. Kaplan–Meier curves. Survival curves for individuals classified as high risk and low risk by (A) the M+BA risk score and (B) the Mayo risk score in both the training and validation data sets.

Table 2. Sensitivity, specificity, positive predictive value (PPV) and negative predictive value (NPV) of the M+BA risk score and Mayo risk score when categorising individuals on likelihood of liver transplant/death in the short term (≤3 years) and long term (>3 years).

Years	n	Risk score	Sensitivity	Specificity	PPV	NPV
≤3 years	23	M+BA	1	0.61	0.42	1
		Mayo	1	0.44	0.33	1
>3 years	27	M+BA	0.50	0.71	0.27	0.83
		Mayo	0.67	0.48	0.33	0.83

of the 152 participants experienced adverse liver-related outcomes (18 liver transplants, 16 deaths). We utilised quantitative MRCP (MRCP+) to derive a prognostic score with high accuracy and then validated the risk score in a separate cohort of patients with PSC. Using data-driven techniques, three predictive metrics were identified including one MRCP+ metric (the proportion of the bile ducts with diameter 3–5 mm) and two serum biochemical markers (AST and total bilirubin). When combined to create a risk score, the M+BA risk score predicted survival with excellent discriminative performance (AUROC = 0.86).

These results highlight the value of MRCP+ as a tool that may support biochemical investigations of this severe and poorly understood disease.

Using quantitatively derived metrics, MRCP+ overcomes the challenges associated with the significant inter-reader variability and subjective interpretation of standard MRCP images, which have thus far frustrated imaging-derived biomarkers for PSC.[18] To date, the clinical risk scores applied in PSC, such as the Mayo risk score, rely predominantly on indirect assessments of biliary health such as blood biomarkers and age. These factors have proven effective at predicting short-term survival in end-stage disease. However, given that biochemical changes only occur in later stages of the disease, the power to discriminate early stages or predict progression before end-stage disease has been limited, as evidenced in this study by the low specificity observed with longer survival times. By directly assessing the biliary tree and reducing the dependence on indirect blood-based biomarkers, the M+BA biomarker correctly identified patients at risk of adverse clinical events.

Given the heterogeneous nature of PSC and its long time course, we propose that the M+BA risk score could form part of a panel of relevant markers, including blood biomarkers, which taken together capture different aspects of the disease. For example, there are emerging technologies that measure liver stiffness using ultrasound-based vibration-controlled transient elastography (VCTE) or magnetic resonance elastography (MRE). Some investigators have assessed the role of measuring liver stiffness in patients with PSC. Both baseline liver stiffness measurement (LSM) and change over time seem to be predictive of liver-related outcomes.[9,10,13,32] These studies report a significant discriminatory ability of LSM values to identify PSC patients with advanced liver fibrosis.[10,13,32] However, studies correlating these measures to clinical outcomes are few, and LSM may be falsely increased at times of increased biliary pressure from choledocholithiasis and cholangitis.[33] Therefore, the proposed M+BA risk score may have a complementary role with MRE or VCTE.

Quantitative MRCP produces a potential suite of 92 metrics. Reducing the number of features of interest to an interpretable number is, therefore, key. This study used data-driven techniques to select the most appropriate metrics. Reassuringly, the measures identified by the stepwise regression process are in excellent agreement with typically clinically recognised features of PSC. For instance, the impact of dilations and ductal wall thickening is captured through the chosen metric of duct diameter. This metric includes proportion of the biliary tree with diameter ranging from 3–5 mm, which is often observed in cases with multi-focal fibrotic strictures, dilations, and reduced branching of the biliary tree. These findings echo previously reported observations of obliteration of the small peripheral ducts, which generates the 'pruned-tree', in patients with more advanced PSC.[34]

Future studies are now needed to further test the derived M+BA risk score in an additional larger cohort, to assess its impact on clinical decision-making and patient outcomes, and to examine how changes to the parameters over time relate to clinical outcomes. Sample size remains a limiting factor when investigating a rare disease over a long-time course. However, the ability to derive an M+BA risk score from retrospective data should help to overcome some of these issues, as many patients with PSC already have long-term MRCP follow-up data. The quantitative and semi-automatic nature of quantitative MRCP will enable the longitudinal progression of these metrics to be monitored and linked, using robust statistical methods, to eventual outcomes. This will help to address a key limitation of risk scores calculated from a baseline assessment, that they fail to account for changes to parameters over time. Although in this study we were able to demonstrate the performance of the M+BA risk score's predictive potential in both the short (≤3 years) and longer (>3 years), future longitudinal studies, ideally following patients up annually until an outcome is reached, should examine whether changes in quantitative MRCP metrics over time continue to predict outcomes. Given that, in many centers, MRCP data are collected annually, and quantitative MRCP analysis can be generated from most 3D MRCP datasets, patients would not be required to undergo additional investigations, thus facilitating this type of longitudinal study. Typically, such changes over time would be assessed by eye; however, the repeatable nature of MRCP+ technology will allow for quantitative assessment of any changes, better informing prognosis at the individual patient level.

In our exploratory analysis, we identified a second model which, derived from the cohort as a whole, included an expanded number of metrics: the proportion of bile ducts with diameter 3–5 mm, the stricture score sum, the absolute maximum diameter of dilatation severity, creatinine, total bilirubin, ALT, and ALK. This second model also demonstrated excellent discriminative performance. Although the lack of a validation cohort is an important caveat for this second expanded model, the additional power of a larger sample size suggests there may be further predictive signal provided by quantitative MRCP metrics to uncover.

A larger population of patients would also address limitations regarding the representativeness of the population.[35] The relatively high number of clinical events over the study period suggests that this population may be one of advanced disease referred to a specialist tertiary center. Future studies could examine then validate the prognostic ability of the M+BA biomarker at different stages of the disease.

Once incorporated into clinical assessment, quantitative assessment of the biliary tree may facilitate the development and evaluation of novel treatment options, which are sorely needed to improve the prognosis of PSC patients. Although MRCP is the current gold-standard assessment tool for PSC, the variable and qualitative nature of the clinical information obtained from traditional MRCP scans makes it difficult to incorporate standard MRCP results into clinical risk scores. In this study, we have demonstrated the excellent sensitivity and specificity of quantitative MRCP metrics utilised within a clinical risk index and we have also shown the additional discriminatory power that the wide range of information contained in an MRCP image can bring when assessing long-term patient outcomes. Indeed, by collapsing the quantitative MRCP metrics to an easily interpretable risk score, while also accounting for traditional biochemical assessments, it may be possible to identify patients for whom more frequent follow up may be beneficial and reassure patients with quiescent disease. Such an outcome would ensure that patients receive the most appropriate care in a timely fashion and facilitate more efficient utilisation of healthcare resources. This is particularly true given that MRCP+ can be applied to already collected 3D MRCP data, allowing the M+BA risk score to be calculated retrospectively.

Conclusions

We generated an objective composite prognostic biomarker, M+BA risk score, that predicts liver transplant or death with greater accuracy than the Mayo risk score. We validated the M+BA risk score in an independent cohort and demonstrated that the predictive power remained high. In doing so, we have shown good evidence for MRCP+ as a novel technology in predicting outcomes for PSC.

Abbreviations

AIC, Akaike Information Criterion; ALP, alkaline phosphatase; ALT, alanine aminotransferase; AST, aspartate aminotransferase; AUROC, area under the receiver operator curve; ERCP, endoscopic retrograde cholangiopancreatography; LSM, liver stiffness measurement; M+BA, MRCP+ Risk Score; MRCP, magnetic resonance cholangiopancreatography; MRCP+, artificial intelligence-enabled quantitative MRCP; MRE, magnetic resonance elastography; MRI, magnetic resonance imaging; PSC, primary sclerosing cholangitis; TE, echo time; TR, repetition time; VCTE, vibration-controlled transient elastography.

Financial support

No external funding was provided for the conduct of this study.

Conflicts of interest

RAT, LY, SM, and CF are employed by Perspectum Ltd. The other authors declare no conflicts of interests.

Please refer to the accompanying ICMJE disclosure forms for further details.

Authors' contributions

Contributed to study concept, design, data-analysis: RV, VA, CK, MG, FA, NC. Statistical design and analysis: AT, SM, LY. Contributed to MRCP+ analysis: CF. Contributed to writing: RV, VA, CK, MG, FA, NC, AT, CF, LY. Contributed to editing: all authors. Approval of the resulting manuscript: all authors.

Data availability statement

The data that support the findings of this study are available on request from the corresponding author (RV). The data are not publicly available owing to privacy restrictions.

Acknowledgements

Perspectum Ltd. provided free access to MRCP+ software and performed statistical analysis for this project through an institutional academic collaborative agreement.

Supplementary data

Supplementary data to this article can be found online at https://doi.org/10.1016/j.jhepr.2023.100834.

References

[1] Hirschfield GM, Karlsen TH, Lindor KD, Adams DH. Primary sclerosing cholangitis. Lancet 2013;382:1587–1599.
[2] Lindor KD, Kowdley KV, Harrison ME, American College of G. ACG clinical guideline: primary sclerosing cholangitis. Am J Gastroenterol 2015;110:646–659. quiz 660.
[3] Chapman MH, Thorburn D, Hirschfield GM, Webster GGJ, Rushbrook SM, Alexander G, et al. British Society of Gastroenterology and UK-PSC guidelines for the diagnosis and management of primary sclerosing cholangitis. Gut 2019;68:1356–1378.
[4] Chapman R, Fevery J, Kalloo A, Nagorney DM, Boberg KM, Shneider B, et al. Diagnosis and management of primary sclerosing cholangitis. Hepatology 2010;51:660–678.
[5] Burak KW, Angulo P, Lindor KD. Is there a role for liver biopsy in primary sclerosing cholangitis? Am J Gastroenterol 2003;98:1155–1158.
[6] de Vries EM, de Krijger M, Farkkila M, Arola J, Schirmacher P, Gotthardt D, et al. Validation of the prognostic value of histologic scoring systems in primary sclerosing cholangitis: an international cohort study. Hepatology 2017;65:907–919.
[7] de Vries EMG, Farkkila M, Milkiewicz P, Hov JR, Eksteen B, Thorburn D, et al. Enhanced liver fibrosis test predicts transplant-free survival in primary sclerosing cholangitis, a multi-centre study. Liver Int 2017;37:1554–1561.
[8] Goode EC, Clark AB, Mells GF, Srivastava B, Spiess K, Gelson WTH, et al. Factors associated with outcomes of patients with primary sclerosing cholangitis and development and validation of a risk scoring system. Hepatology 2019;69:2120–2135.
[9] Eaton JE, Sen A, Hoodeshenas S, Schleck CD, Harmsen WS, Gores GJ, et al. Changes in liver stiffness, measured by magnetic resonance elastography, associated with hepatic decompensation in patients with primary sclerosing cholangitis. Clin Gastroenterol Hepatol 2020;18:1576–1583.e1571.
[10] Cazzagon N, Lemoinne S, El Mouhadi S, Trivedi PJ, Gaouar F, Kemgang A, et al. The complementary value of magnetic resonance imaging and vibration-controlled transient elastography for risk stratification in primary sclerosing cholangitis. Am J Gastroenterol 2019;114:1878–1885.
[11] Majoie CB, Reeders JW, Sanders JB, Huibregtse K, Jansen PL. Primary sclerosing cholangitis: a modified classification of cholangiographic findings. Am J Roentgenol 1991;157:495–497.
[12] Kim WR, Therneau TM, Wiesner RH, Poterucha JJ, Benson JT, Malinchoc M, et al. A revised natural history model for primary sclerosing cholangitis. Mayo Clin Proc 2000;75:688–694.
[13] Corpechot C, Gaouar F, El Naggar A, Kemgang A, Wendum D, Poupon R, et al. Baseline values and changes in liver stiffness measured by transient elastography are associated with severity of fibrosis and outcomes of patients with primary sclerosing cholangitis. Gastroenterology 2014;146:970–979. quiz e915–76.
[14] Karlsen TH, Folseraas T, Thorburn D, Vesterhus M. Primary sclerosing cholangitis – a comprehensive review. J Hepatol 2017;67:1298–1323.
[15] Ruiz A, Lemoinne S, Carrat F, Corpechot C, Chazouilleres O, Arrive L. Radiologic course of primary sclerosing cholangitis: assessment by three-dimensional magnetic resonance cholangiography and predictive features of progression. Hepatology 2014;59:242–250.
[16] Chapman RW. Malignancy in primary sclerosing cholangitis: bile duct, liver, and colon. Clin Liver Dis (Hoboken) 2014;3:83–85.
[17] Ismail MF, Hirschfield GM, Hansen B, Tafur M, Elbanna KY, Goldfinger MH, et al. Evaluation of quantitative MRCP (MRCP+) for risk stratification of primary sclerosing cholangitis: comparison with morphological MRCP, MR elastography, and biochemical risk scores. Eur Radiol 2022;32:67–77.

[18] Zenouzi R, Liwinski T, Yamamura J, Weiler-Normann C, Sebode M, Keller S, et al. Follow-up magnetic resonance imaging/3D-magnetic resonance cholangiopancreatography in patients with primary sclerosing cholangitis: challenging for experts to interpret. Aliment Pharmacol Ther 2018;48:169–178.
[19] Selvaraj EA, Ba-Ssalamah A, Poetter-Lang S, Ridgway GR, Brady JM, Collier J, et al. A quantitative magnetic resonance cholangiopancreatography metric of intrahepatic biliary dilatation severity detects high-risk primary sclerosing cholangitis. Hepatol Commun 2022;6:795–808.
[20] Moons KG, Royston P, Vergouwe Y, Grobbee DE, Altman DG. Prognosis and prognostic research: what, why, and how? BMJ 2009;338:b375.
[21] Royston P, Moons KG, Altman DG, Vergouwe Y. Prognosis and prognostic research: developing a prognostic model. BMJ 2009;338:b604.
[22] Moons KG, Altman DG, Vergouwe Y, Royston P. Prognosis and prognostic research: application and impact of prognostic models in clinical practice. BMJ 2009;338:b606.
[23] Cristoferi L, Porta M, Bernasconi DP, Leonardi F, Gerussi A, Mulinacci G, et al. A quantitative MRCP-derived score for medium-term outcome prediction in primary sclerosing cholangitis. Dig Liver Dis 2023;55:373–380.
[24] Cazzagon N, El Mouhadi S, Vanderbecq Q, Ferreira C, Finnegan S, Lemoinne S, et al. Quantitative magnetic resonance cholangiopancreatography metrics are associated with disease severity and outcomes in people with primary sclerosing cholangitis. JHEP Rep 2022;4:100577.
[25] www.cdc.gov/nchs/ndi/index.htm?CDC_AA_refVal=https%3A%2F%2Fwww.cdc.gov%2Fnchs%2Fndi.htm. [Accessed 1 January 2023].
[26] Goldfinger MH, Ridgway GR, Ferreira C, Langford CR, Cheng L, Kazimianec A, et al. Quantitative MRCP imaging: accuracy, repeatability, reproducibility, and cohort-derived normative ranges. J Magn Reson Imaging 2020;52:807–820.
[27] Cheng LNR, Langford C, Shumbayawonda E, Dennis A, Mavar M, et al. GI05-A7 Characterization of abnormalities in the biliary system by quantitative MRCP imaging: a repeatability and reproducibility study RSNA 2021. https://www.rsna.org/-/media/Files/RSNA/Annual-meeting/Future-and-past-meetings/RSNA2021_Meeting_Program.ashx.
[28] de Vries EM, Wang J, Williamson KD, Leeflang MM, Boonstra K, Weersma RK, et al. A novel prognostic model for transplant-free survival in primary sclerosing cholangitis. Gut 2018;67:1864–1869.
[29] Yang Y, Zou H. A cocktail algorithm for solving the elastic net penalized Cox's regression in high dimensions. Stat Interf 2013;6:167–173. Available from: https://www.math.mcgill.ca/yyang/resources/papers/SII_fastcox.pdf. [Accessed 19 January 2023].
[30] Johnston R, Jones K, Manley D. Confounding and collinearity in regression analysis: a cautionary tale and an alternative procedure, illustrated by studies of British voting behaviour. Qual Quant 2018;52:1957–1976.
[31] Kim WR, Poterucha JJ, Wiesner RH, LaRusso NF, Lindor KD, Petz J, et al. The relative role of the Child-Pugh classification and the Mayo natural history model in the assessment of survival in patients with primary sclerosing cholangitis. Hepatology 1999;29:1643–1648.
[32] Tafur M, Cheung A, Menezes RJ, Feld J, Janssen H, Hirschfield GM, et al. Risk stratification in primary sclerosing cholangitis: comparison of biliary stricture severity on MRCP versus liver stiffness by MR elastography and vibration-controlled transient elastography. Eur Radiol 2020;30:3735–3747.
[33] Vuppalanchi R, Lindor K. Magic wand and crystal ball for primary sclerosing cholangitis. Gastroenterology 2014;146:890–892.
[34] Khoshpouri P, Habibabadi RR, Hazhirkarzar B, Ameli S, Ghadimi M, Ghasabeh MA, et al. Imaging features of primary sclerosing cholangitis: from diagnosis to liver transplant follow-up. Radiographics 2019;39:1938–1964.
[35] Altman DG, Vergouwe Y, Royston P, Moons KG. Prognosis and prognostic research: validating a prognostic model. BMJ 2009;338:b605.

Journal of Hepatology, Volume *5*

Supplemental information

A composite score using quantitative magnetic resonance cholangiopancreatography predicts clinical outcomes in primary sclerosing cholangitis

Raj Vuppalanchi, Vijay Are, Alison Telford, Liam Young, Sofia Mouchti, Carlos Ferreira, Carla Kettler, Mark Gromski, Fatih Akisik, and Naga Chalasani

Supplementary materials

A composite score using quantitative magnetic resonance cholangiopancreatography predicts clinical outcomes in primary sclerosing cholangitis

Raj Vuppalanchi, Vijay Are, Alison Telford, Liam Young, Sofia Mouchti, Carlos Ferreira, Carla Kettler, Mark Gromski, Fatih Akisik, Naga Chalasani

Table of contents

Supplementary results 2

Derivation of an extended M+B Risk score 2

Diagnostic performance of extended M+B Risk score 2

Fig. S1. Forest plot of extended M+B Risk score metrics 3

Fig. S2. Receiver operator curve of extended M+B Risk score 4

Fig. S3. Kaplan-Meier Curve of extended M+B Risk score 5

Table S1. Summary statistics of quantitative MRCP and biochemical metrics for patients who survived transplant-free or had a clinical event. 6

Table S2. Hazard ratios of a unit increase in the quantitative MRCP and blood metrics chosen by the Stepwise Cox regression model. 9

Supplementary CTAT Table 9

Software 9

Details of the corresponding methods author for the manuscript 9

Supplementary results

Derivation of an extended M+B Risk score

A secondary analysis was performed using the same statistical methodology on a pooled cohort consisting of both the test and validation cohorts. The stepwise regression method for Cox's Proportional Hazard model returned the Risk Score of quantitative MRCP and blood metrics (extended M+B Risk Score). In the larger cohort, seven metrics were included in the extended M+B model (Fig. S1) including: proportion of ducts with diameter 3-5 mm (%), the total stricture score, the maximum absolute severity of dilation (mm), serum total bilirubin (mg/dL), serum alkaline phosphatase (iU), serum ALT (iU), and serum creatinine (mg/dL). A Cox proportional hazard model used these metrics to predict survival through the formula:

$$\ln\left\{\frac{h(t)}{h_0(t)}\right\} = 1.065 \times \text{Proportion of the biliary tree with diameter 3-5mm} \; +$$

$$1.232 \times \text{Maximum absolute severity of dilations} + \\ 0.957 \times \text{Total stricture score} + \\ 3.571 \times \text{Total Bilirubin} \; + \\ 4.842 \; \times \; \text{Creatinine} + \\ 1.129 \; \times \text{ALK} + \\ 0.446 \; \times \; \text{ALT}$$

A unit increase in each of the metrics significantly increased the hazard ration of a clinical event for each metric. The changes in hazard ratio for a unit increase are shown in Table S2. Creating a composite extended M+B Risk Score using the model coefficients was found to significantly increase the hazard ratio of an event by tbc (Fig. S2).

Diagnostic performance of extended M+B Risk score

The extended M+B Risk Score had excellent discrimination of transplant-free survival with Harrell's C-statistic of 0.86 (95% CI: 0.78, 0.93; Fig. S3). The extended M+B score had marginally higher diagnostic performance than the M+BA score derived on a subset of the data. However, this was not significant and the M+BA score maintained excellent diagnostic performance (C-statistic 0.84 [95% CI: 0.77 – 0.91]). Using the Youden's index for the training dataset, a threshold for classifying high-risk individuals using the extended M+B Risk Score was established as those with an extended M+B Risk score of greater than 4.63.

Applying this threshold, participants classified as high-risk by the extended M+B Risk Score had significantly higher hazard ratio of experiencing death or liver transplant (11.66 [95% CI: 5.04, 26.97]) (Fig. S4). Participants classified as high-risk by the Mayo risk score had hazard ratios of 3.27 (95% CI: 1.35, 7.91) using the previously published threshold. The extended M+B Risk Score was found to be a better fit for explaining transplant-free survival with a AIC of 266.67 compared to the AIC of 301.68 for the Mayo Risk Score.

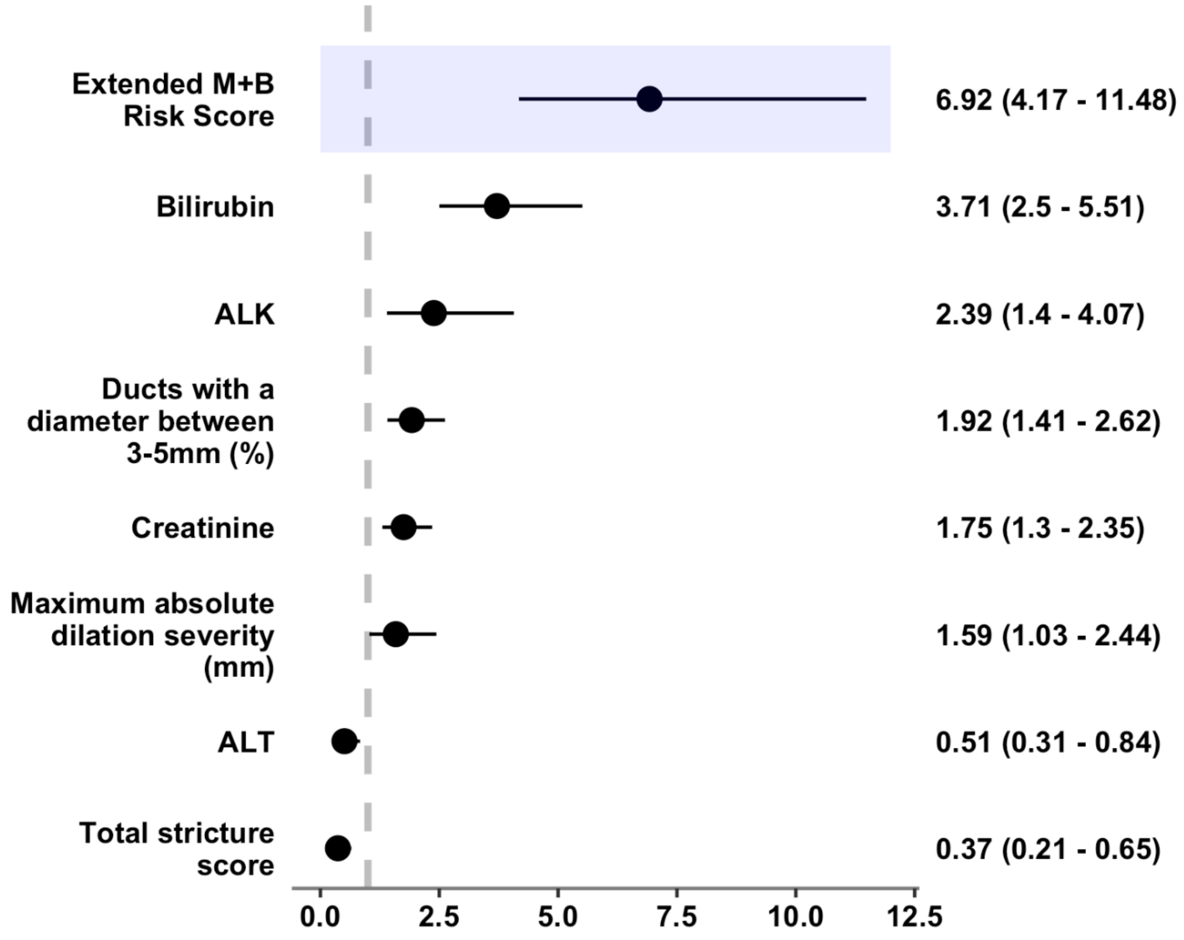

Fig. S1: Forest plot of extended M+B Risk score metrics. Shaded box: Hazard Ratio and corresponding 95% confidence interval of the extended M+B Risk score. Below the shaded box: Hazard Ratios and corresponding 95% confidence interval of the quantitative MRCP and blood metrics chosen by the Stepwise Cox regression model. All the metrics were standardised as z-scores.

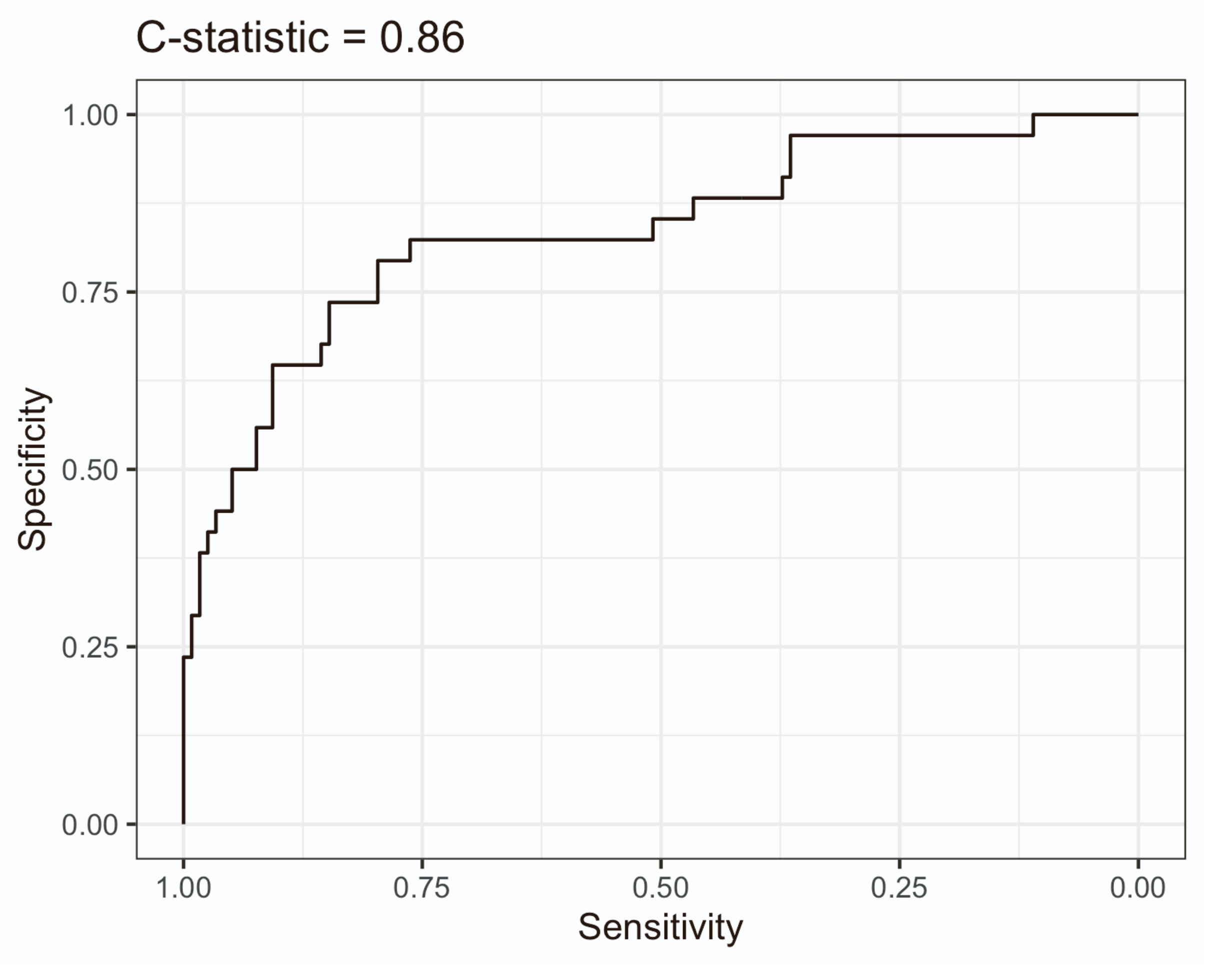

Fig. S2: Receiver operator curve of extended M+B Risk score. Diagnostic accuracy of the extended M+B Risk of discriminating death or liver transplant in the combined cohort.

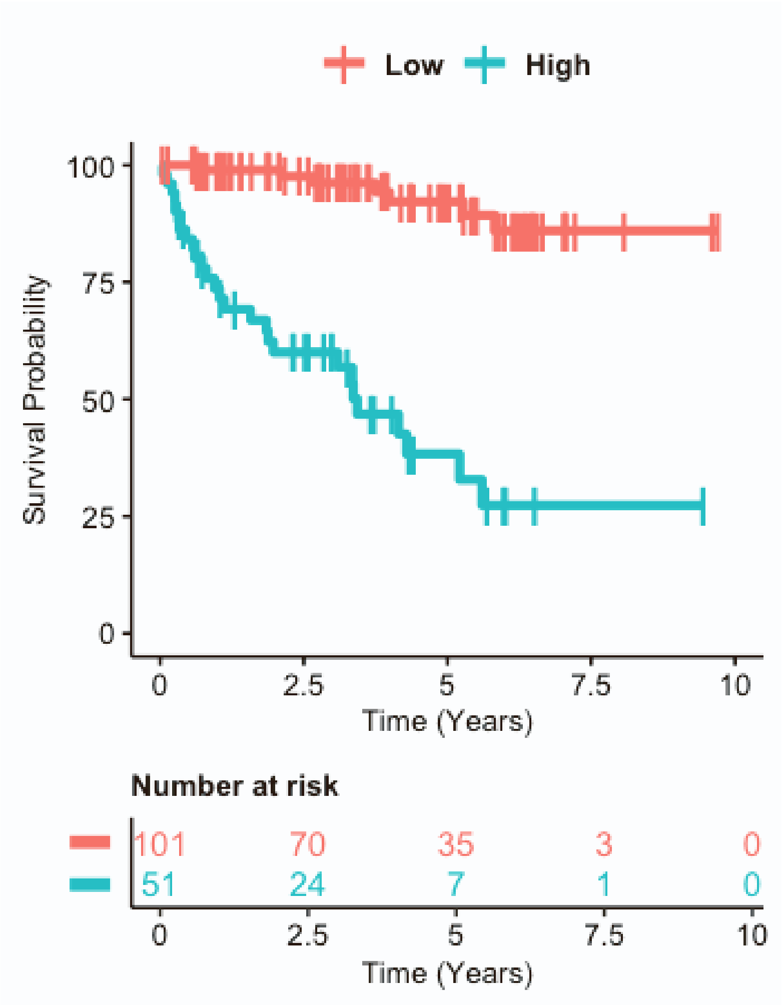

Fig. S3. Kaplan-Meier Curve of extended M+B Risk score. Survival curves for individuals classified as high-risk and low-risk by the extended M+B Risk Score the combined data set.

Table S1. Summary statistics of quantitative MRCP and biochemical metrics for patients who survived transplant-free or had a clinical event. Metrics in italics were those that were included in the stepwise model selection. Data are presented as mean (SD) or median (IQR) for continuous variables, and as numbers and percentages (%) for categorical data.

Metric	All	No Liver Transplant/ Death	Liver Transplant/ Death	p-value*
**Quantitative MRCP**				
Number of ducts (n)	64 (33, 102)	66 (35, 101)	52 (21, 102)	0.6
Number of strictures (n)	7 (3, 12)	7 (3, 14)	8 (2, 12)	0.7
Number of dilations (n)	14 (6, 25)	14 (6, 26)	16 (5, 23)	0.7
Number of strictures with a length < 3 mm (n)				0.7
0	98 (64%)	79 (67%)	19 (56%)	
1	38 (25%)	26 (22%)	12 (35%)	
2	12 (7.9%)	10 (8.5%)	2 (5.9%)	
3	3 (2%)	2 (1.7%)	1 (2.9%)	
6	1 (0.7%)	1 (0.8%)	0 (0%)	
Number of strictures with a length between 3-10 mm	5.0 (2.0, 9.2)	5.0 (2.0, 10.0)	5.0 (1.3, 9.0)	0.7
Number of ducts containing one or more stricture (n)	6.0 (3.0, 10.0)	6.0 (3.0, 10.0)	6.0 (2.0, 10.0)	0.7
Number of ducts containing one or more dilation (n)	12 (5, 19)	12 (5, 20)	12 (4, 18)	0.7
Number of ducts containing one or more stricture and dilation (n)	4.0 (1.0, 7.0)	4.0 (1.0, 7.0)	4.0 (1.0, 6.0)	0.7
Number of ducts containing one or more stricture OR dilation (n)	14 (6, 22)	14 (8, 23)	14 (5, 22)	0.7
Total length of ducts (mm)	1059 (549, 1912)	1093 (568, 2011)	829 (329, 1778)	0.3
Total length of strictures (mm)	52 (21, 95)	52 (23, 97)	52 (16, 88)	0.6
Total length of dilations (mm)	86 (36, 163)	85 (38, 161)	87 (28, 160)	0.7
Total length of strictures and dilations (mm)	150 (58, 262)	152 (61, 259)	143 (37, 256)	0.7

Proportion of the biliary tree with diameter between 5-7 mm (%)	0.029 (0.013, 0.048)	0.029 (0.012, 0.045)	0.030 (0.017, 0.057)	0.3
Proportion of the biliary tree with diameter between 7-9 mm (%)	0.004 (0.000, 0.012)	0.003 (0.000, 0.011)	0.008 (0.003, 0.018)	0.2
Proportion of bile ducts with a diameter between 3-5 mm (%)	19 (13, 28)	18 (12, 27)	24 (16, 33)	0.029
Proportion of bile ducts with a diameter between 5-7 mm (%)	1.53 (0.00, 4.00)	1.47 (0.00, 3.81)	1.62 (0.00, 4.59)	0.7
Maximum length of ducts (mm)	80 (67, 100)	82 (67, 100)	77 (59, 92)	0.7
Maximum absolute severity of strictures (mm)	2.24 (1.71, 3.33)	2.23 (1.70, 3.34)	2.28 (1.79, 3.20)	0.9
Maximum absolute severity of dilatations (mm)	3.82 (2.54, 5.11)	3.59 (2.56, 5.04)	4.18 (2.58, 5.83)	0.7
Maximum relative severity of dilatations (%)	1.30 (0.93, 1.88)	1.28 (0.93, 1.80)	1.42 (0.99, 2.00)	0.7
Sum of the severity score for strictures	23 (9, 41)	23 (10, 42)	21 (6, 28)	0.6
Sum of severity scores for dilatations	58 (24, 109)	57 (26, 109)	66 (18, 104)	0.9
Sum of absolute severity of strictures (mm)	11 (4, 21)	11 (5, 21)	13 (3, 20)	0.7
Sum of absolute severity of dilatations	26 (10, 48)	24 (10, 47)	30 (12, 47)	0.9
Sum of relative severity of strictures (%)	3.2 (1.3, 5.5)	3.1 (1.5, 5.5)	3.4 (0.7, 5.4)	0.7
Sum of relative severity of dilatations (%)	9 (4, 17)	9 (4, 18)	11 (3, 16)	0.7
**Biochemical**				
Sodium	4.927 (4.913, 4.934)	4.927 (4.913, 4.934)	4.920 (4.905, 4.927)	0.058
Creatinine	-0.21 (-0.37, -0.02)	-0.21 (-0.36, -0.01)	-0.25 (-0.43, -0.02)	0.7
Albumin	1.34 (1.22, 1.42)	1.36 (1.28, 1.44)	1.18 (1.01, 1.31)	<0.001

Bilirubin	0.10 (-0.36, 0.93)	0.00 (-0.51, 0.41)	1.36 (0.14, 1.98)	<0.001
ALT	4.03 (3.33, 4.54)	3.97 (3.27, 4.54)	4.30 (3.74, 4.53)	0.3
AST	3.95 (3.40, 4.44)	3.87 (3.33, 4.227)	4.30 (4.03, 4.77))	0.001
ALK	5.46 (4.85, 6.08)	5.38 (4.80, 5.93)	6.02 (5.31, 6.26)	0.018
Platelet Count	5.39 (5.06, 5.65)	5.44 (5.16, 5.65)	5.37 (4.92, 5.62)	0.7
INR	1.09 (1.01, 1.23)	1.08 (1.00, 1.18)	1.19 (1.07, 1.42)	0.022
**Other**				
Mayo Risk Score	0.16 (-0.36, 1.16)	0.08 (-0.51, 0.61)	1.37 (0.59, 2.39)	<0.001
Mayo Risk Score Categorical				<0.001
High	16 (11%)	5 (4.2%)	11 (32%)	
Intermediate	78 (51%)	61 (52%)	17 (50%)	
Low	58 (38%)	52 (44%)	6 (18%)	

Table S2. Hazard ratios of a unit increase in the quantitative MRCP and blood metrics chosen by the Stepwise Cox regression model. All blood metrics are log transformed prior to assessment.

Metric*	HR (95% CI)
Proportion of ducts with diameter 3-5 mm	1.06 (1.03 – 1.10)
Maximum absolute severity of dilation	1.23 (1.01 – 1.50)
Total stricture score	0.96 (0.93 – 0.98)
serum total bilirubin	3.57 (2.43 – 5.24)
ALK	3.09 (1.55 – 6.18)
Creatinine	4.84 (2.11 – 11.13)
ALT	0.45 (0.25 – 0.81)

Supplementary CTAT Table

Software

Software name	Manufacturer	Version
**MRCP++**	**Perspectum Ltd, Oxford, UK**	**V1.0.0**

Details of the corresponding methods author for the manuscript

Raj Vuppalanchi, MD
Professor of Medicine,
Division of Gastroenterology and Hepatology,
Indiana University School of Medicine,
702 Rotary Circle, Suite 225,
Indianapolis, IN 46202, USA
rvuppala@iu.edu
